# Loss of the Mechanistic Target of Rapamycin Complex 1 Causes a Lethal Alpha-1 Antitrypsin Deficiency–Associated Liver Disease

**DOI:** 10.1016/j.jcmgh.2026.101824

**Published:** 2026-06-04

**Authors:** Lisa Bewersdorf, Sophie Haber, Ines Volkert, Katharina Remih, Annika Gross, Christian Preisinger, Linda Kroll, Thorsten Cramer, Nadine Therese Gaisa, Sophie Leypold, Ulrike Rolle-Kampczyk, Nicola Brunetti-Pierri, Martin von Bergen, Florian Rosenberger, Caroline Weiss, Nurdan Guldiken, Pavel Strnad

**Affiliations:** 1 Department of Medicine III, University Hospital Aachen, Aachen, Germany; 2 Interdisciplinary Center for Clinical Research, University Hospital Aachen, Aachen, Germany; 3 Department of General, Visceral and Transplantation Surgery, University Hospital Aachen, Aachen, Germany; 4 Institute of Pathology, University Hospital Aachen, Aachen, Germany; 5 Department of Molecular Systems Biology, Helmholtz-Centre for Environmental Research-UFZ, Leipzig, Germany; 6 Telethon Institute of Genetics and Medicine (TIGEM), Pozzuoli, Naples, Italy; 7 Department of Translational Medicine, Federico II University, Naples, Italy; 8 Scuola Superiore Meridionale (SSM, School of Advanced Studies), Genomics and Experimental Medicine Program, University of Naples Federico II, Naples, Italy; 9 Department of Proteomics and Signal Transduction, Max Planck Institute of Biochemistry, Martinsried, Germany; 10 Department of Gastroenterology and Hepatology, University Hospital Lausitz-Carl Thiem, Cottbus, Germany

**Keywords:** Hyperammonemia, Liver Zonation, Metabolic Reprogramming, Proteostasis

## Abstract

**Background & Aims:**

*SERPINA1* mutations cause retention of the otherwise secreted alpha-1 antitrypsin and lead to the proteotoxic alpha-1 antitrypsin deficiency–related liver disease. As mechanistic target of rapamycin is a key coordinator of proteostasis, we studied its role in alpha-1 antitrypsin deficiency–related liver disease.

**Methods:**

PiZ mice overexpressing the characteristic *SERPINA1* mutation were mated with rodents harboring a hepatocyte specific–ablation of the interaction partners regulatory-associated protein of mechanistic target of rapamycin or rapamycin-insensitive companion of mammalian target of rapamycin, corresponding to mechanistic target of rapamycin complexes 1 or 2, or with mice lacking mechanistic target of rapamycin. Serum proteomics, liver bulk proteomics, spatial proteomics, and metabolomics were applied to characterize molecular and metabolic alterations.

**Results:**

At 2 months of age, PiZ-mTOR^Δhep^ and PiZ-Raptor^Δhep^ but not PiZ-Rictor^Δhep^ mice showed signs of increased liver injury and mortality despite diminished hepatic alpha-1 antitrypsin accumulation. PiZ-Raptor^Δhep^ animals displayed increased levels of the proapoptotic protein C/EBP homologous protein, but C/EBP homologous protein ablation did not rescue the phenotype. Serum proteomics revealed no signs of advanced synthetic liver failure but immature hepatocellular products. Liver bulk proteomics and small metabolite measurement demonstrated a metabolic reprogramming of PiZ-Raptor^Δhep^ mice. Spatial proteomics revealed alterations in liver zonation with increased ammonia levels as the likely cause of death in PiZ-Raptor^Δhep^ animals.

**Conclusions:**

In summary, in alpha-1 antitrypsin deficiency–related proteotoxic liver injury, regulatory-associated protein of mechanistic target of rapamycin preserves a liver zonation, thereby protecting from lethal metabolic dysregulation.

## Graphical abstract

**Figure:**
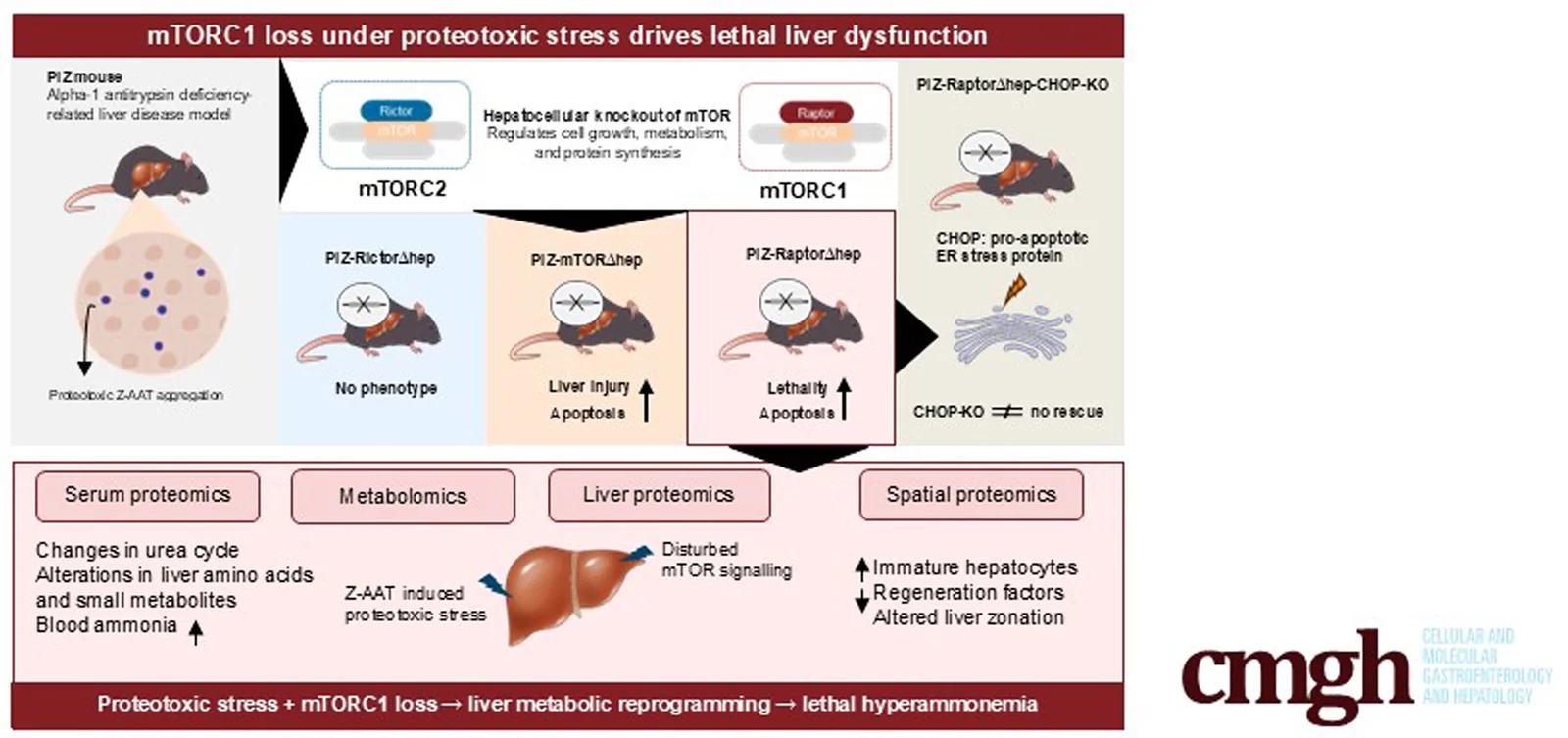

**Summary**

Loss of mechanistic target of rapamycin complex 1 (regulatory-associated protein of mechanistic target of rapamycin) in mice with alpha-1 antitrypsin deficiency triggered liver injury and lethal metabolic dysregulation. Findings display the key role of regulatory-associated protein of mechanistic target of rapamycin in maintaining liver zonation and protecting against alpha-1 antitrypsin deficiency–related liver disease.

**What You Need to Know**

**Background**

Alpha-1 antitrypsin deficiency (AATD) is a proteotoxic liver disease caused by retention of mutated alpha-1 antitrypsin. Mechanistic target of rapamycin (mTOR) with its complexes mechanistic target of rapamycin complex 1/2 constitutes a key regulator of proteostasis.

**Impact**

This study identifies a protective role of mechanistic target of rapamycin complex 1 in a mouse model of AATD liver disease, linking liver zonation to metabolic homeostasis and highlighting potential risks of mTOR inhibition.

**Future Directions**

Future studies should delineate the complex interaction between mTOR inhibition and liver disease, as well as to determine the impact of the commonly used mTOR inhibitors on human AATD.

Alpha-1 antitrypsin (AAT) is an acute-phase protein that is predominantly produced in the endoplasmic reticulum of hepatocytes and is secreted into the blood.^1^^,^^2^ Alpha-1 antitrypsin deficiency (AATD) is an inherited disease caused by mutations in the AAT-encoding gene *SERPINA1*.^1^ PiZ, as the most prevalent disease-causing *SERPINA1* variant, arises due to a replacement of a single amino acid (mutant alpha-1 antitrypsin with replacement of the amino acid glutamic acid 342 to lysine [Z-AAT]) that leads to AAT polymerization, hepatocellular retention, and subsequent gain of function liver toxicity.^1^^,^^2^ Homozygous PiZ occurrence (termed PiZZ genotype) can lead to the development of both pediatric and adult AATD-associated liver disease (AATD-LD). In the former, children are susceptible to prolonged neonatal cholestasis, and 2% to 3% of them develop end-stage liver disease, whereas the adult form typically manifests in the fourth or fifth decade and is seen in 20% to 35% of carriers, who display significant liver fibrosis.^3^^,^^4^ The PiZZ genotype is the leading genetic cause of pediatric liver transplantation and confers 20 times higher risk of liver cirrhosis in adults.^3^^,^^4^ Approximately 15% of Z-AAT is secreted into the blood, but a majority is retained in the liver.^5^ Around 70% of the produced Z-AAT is degraded by autophagy or endoplasmic reticulum (ER)–associated protein degradation, and 15% accumulates as polymers.^6^^,^^7^ In adults, increased intrahepatic Z-AAT retention is associated with more advanced liver fibrosis.^8^^,^^9^ In line with that, the pace of Z-AAT degradation in patient-derived fibroblasts correlated with the extent of AATD-LD.^10^ The notion that AATD-LD constitutes a proteotoxic disorder was demonstrated by studies in mice overexpressing human PiZ (PiZ mice) that serve as a model of AATD-LD and most recently by human single-cell proteomic data.^11^ Accordingly, activation of autophagy, by both pharmaceutical and genetic means, led to improvement of liver phenotype, and hepatocytes with higher inclusion load displayed signs of ER stress.^11^, ^12^, ^13^, ^14^ Both overexpression of the mechanistic target of rapamycin (mTOR) downstream target transcription factor EB as well as a treatment with the mTOR inhibitor rapamycin was beneficial in PiZ mice,^13^^,^^15^ thus pointing to mTOR signaling as a key proteostatic regulator in AAT-LD.

mTOR is a conserved serine/threonine protein kinase that is the core of 2 distinct protein complexes named mTOR complex 1 (mTORC1) and complex 2 (mTORC2).^16^^,^^17^ Although both complexes share several proteins such as mTOR, they also possess specific constituents like regulatory-associated protein of mTOR (RAPTOR) for mTORC1 and rapamycin-insensitive companion of mammalian target of rapamycin (RICTOR) for mTORC2.^18^ mTOR activity is influenced by multiple factors, including environmental stress, amino-acid sensing, energy status or insulin, and growth factors, and constitutes 1 of the most important regulators of metabolism, cell growth, anabolism, and catabolism.^19^^,^^20^ mTORC1 controls protein synthesis through the phosphorylation of downstream targets eukaryotic translation initiation factor 4E-binding protein 1 and p70S6 kinase 1^21^, ^22^, ^23^ and decreases overall protein degradation by the ubiquitin–proteasome system. mTORC2 is mainly known for its regulatory effect on survival and proliferation.^17^^,^^24^, ^25^, ^26^ Importantly, both mTORCs constitute important pharmaceutical targets,^27^^,^^28^ and mTOR inhibitors are regularly used in the clinic.^29^ Additionally, recent advances in RNA interference enable a hepatocyte-specific silencing of genes of interest such as mTOR components.^30^ To investigate the therapeutic potential of such an approach, we assessed the relevance of hepatocellular mTOR signaling in AATD-LD by cross-breeding PiZ mice with animals lacking either mTOR or its major constituents RAPTOR or RICTOR and systematically studied the downstream consequences. To further elucidate the importance of cell death in this setting, a mating with C/EBP homologous protein (CHOP)-deficient animals was performed, given its disease-promoting effect in experimental AATD-LD.^31^

## Results

### Increased Hepatocellular Injury in PiZ-mTOR^Δhep^ Mice

**Figure 1:**
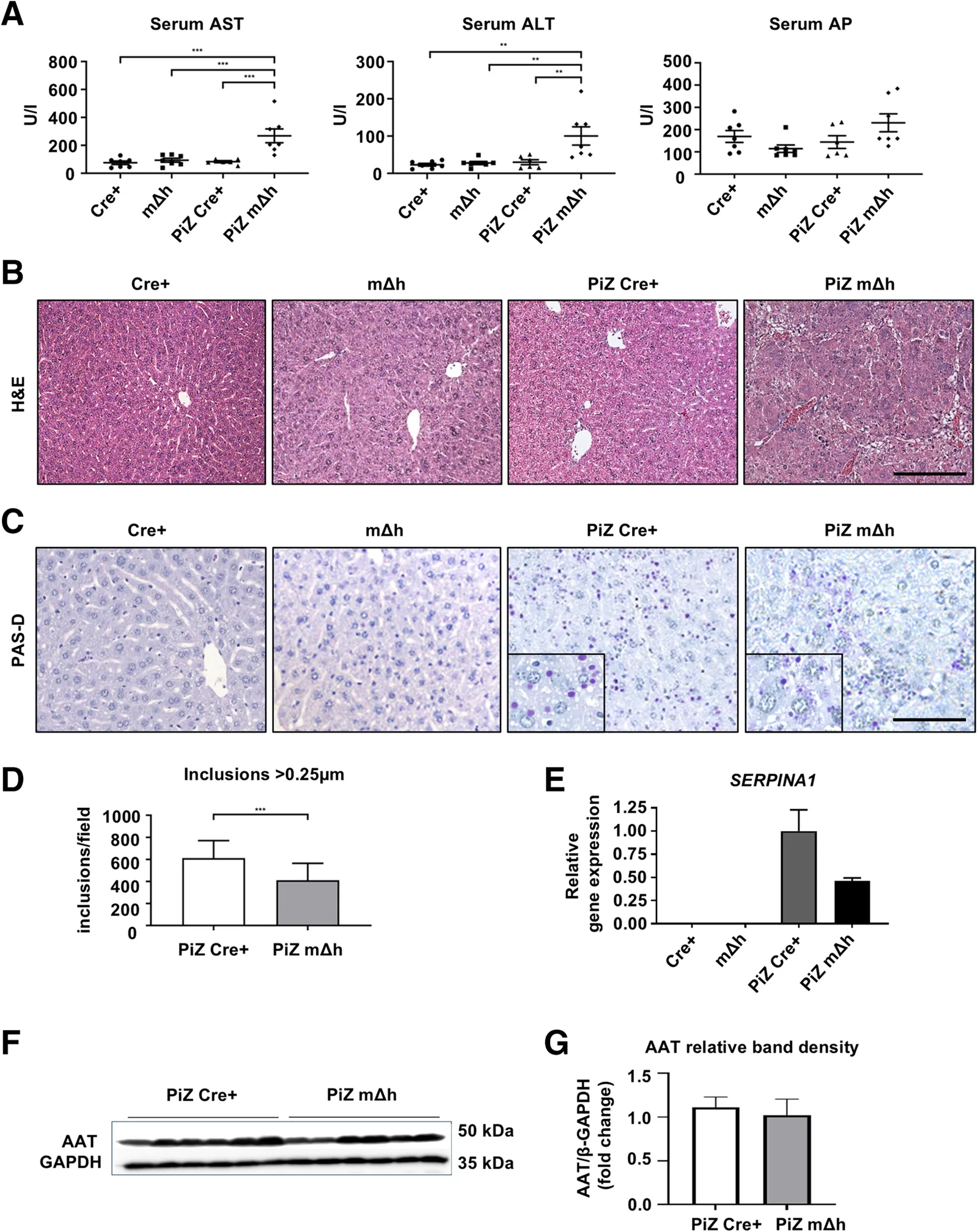
**Increased liver injury in 2-month-old PiZ mTORΔhep mice.** (*A*) Liver enzymes AST, alanine aminotransferase (ALT), and alkaline phosphatase (AP) in sera of 2-month-old Cre+ mice, mTOR hepatocyte-specific knockout mice (mΔhep), PiZ and Cre+ mice (PiZ Cre+), and triple transgenic animals (PiZ mΔhep). (*B*) Representative hematoxylin and eosin (H&E; scale bar, 200 μm) and (*C*) PAS-D stainings (scale bar, 100 μm). (*D*) Number of inclusions with a size bigger than 0.25 μm counted with ImageJ. (*E*) qRT-PCR–based relative quantification of *SERPINA1* (AAT gene) levels with ribosomal protein L7 (L7) as an internal control. Messenger RNA (mRNA) levels in PiZ Cre+ were arbitrarily set as 1, and the levels in other groups are presented as ratios. (*F*) Immunoblot of total liver lysates with an antibody against AAT and β-actin as a loading control and the corresponding morphometric quantification (*G*). Results are shown as mean ± standard error of the mean (*A*, n = 7; *B–E*, n = 5; *F* and *G*, n = 6). Asterisks indicate ∗∗*P* ≤ .01 and ∗∗∗*P* ≤ .001. GAPDH, glyceraldehyde 3-phosphate dehydrogenase.

**Figure 2:**
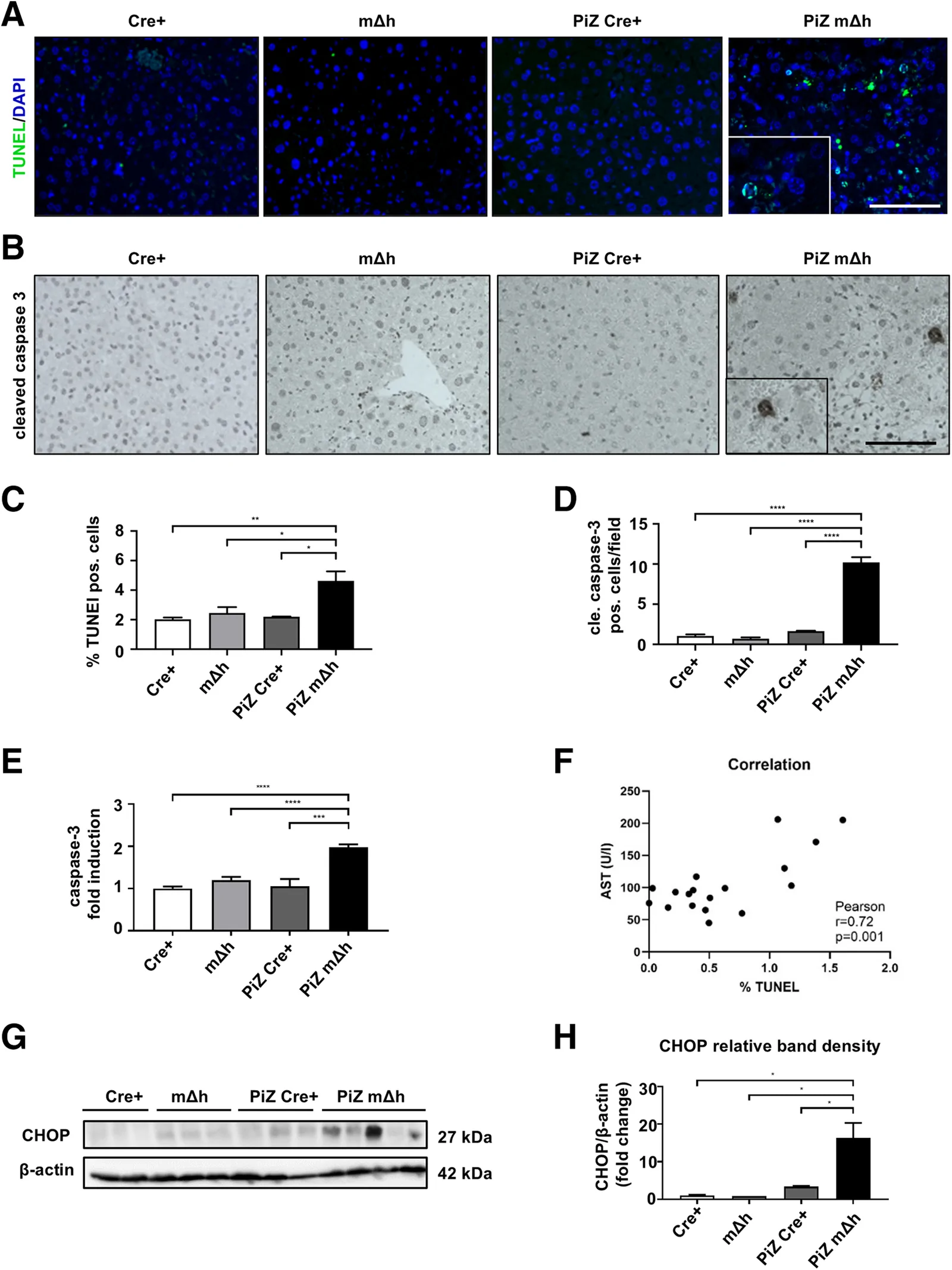
**Increased apoptosis and CHOP levels in 2-month-old PiZ mTORΔhep mice.** (*A*) TUNEL and (*B*) immunohistochemical staining for cleaved caspase-3 in liver tissues from 2-month-old Cre+ mice, mTOR hepatocyte-specific knockdown mice (mΔhep), PiZ and Cre+ mice (PiZ Cre+), and triple transgenic animals (PiZ mΔhep). Positive cells are indicated by *green, fluorescent* nuclei (*A*) and by *brown staining* of the cytoplasm (*B*). Scale bar, 100 μm. (*C* and *D*) Quantification was performed with ImageJ. (*E*) Cleaved caspase-3 assay from whole liver tissue homogenates. (*F*) Pearson correlation analysis between liver injury marker (AST) and the intensity of TUNEL staining. (*G*) Immunoblot of whole liver lysates for CHOP protein levels with β-actin as a loading control, and the corresponding morphometric quantification (*H*). Results are shown as mean ± standard error of the mean (*A–D*, n = 5–6; *E*, n = 4–5; *F* and *G*, n = 3–5). Asterisks indicate ∗*P* ≤ .05; ∗∗*P* ≤ .01; ∗∗∗*P* ≤ .001; ∗∗∗∗*P* ≤ .0001.

To investigate the relevance of hepatocellular mTOR in AATD-LD, we crossbred PiZ mice with hepatocyte-specific mTOR knockouts (KOs) (mTOR^Δhep^). At 2 months of age, PiZ-mTOR^Δhep^ mice displayed significantly elevated aspartate aminotransferase (AST) and alanine aminotransferase serum levels, but no changes in alkaline phosphatase (Figure 1*A*). Liver pathology revealed enlarged hepatocellular nuclei and cytoplasmic clearing in some PiZ mTOR^Δhep^ hepatocytes, whereas mTOR^Δhep^ livers did not show alterations (Figure 1*B*). Periodic acid-Schiff diastase (PAS-D) staining detected smaller amounts of larger AAT globules (Figure 1*C* and *D*), whereas levels of *SERPINA1* messenger RNA and AAT protein levels were unaffected between PiZ and PiZ-mTOR^Δhep^ animals (Figure 1*E–G*). Terminal deoxynucleotidyl transferase dUTP nick end labeling (TUNEL) demonstrated a significant increase of TUNEL-positive nuclei in PiZ-mTOR^Δhep^ livers (Figure 2*A*) that was in line with increased cleaved caspase (Figure 2*B*–*E*). Pearson correlation analysis revealed a positive association between the serologic liver injury marker AST and the amount of TUNEL-positive cells (Figure 2*F*). Moreover, PiZ-mTOR^Δhep^ livers harbored elevated levels of the proapoptotic transcription factor CHOP (Figure 2*G* and *H*). Taken together, these findings show that hepatocyte-specific loss of mTOR aggravates liver cell death injury induced by Z-AAT.

### Hepatocellular Loss of Regulatory-Associated Protein of Mechanistic Target of Rapamycin (Mechanistic Target of Rapamycin Complex 1), but not Rapamycin-Insensitive Companion of Mammalian Target of Rapamycin, Promotes Liver Injury in PiZ Mice

**Figure 3:**
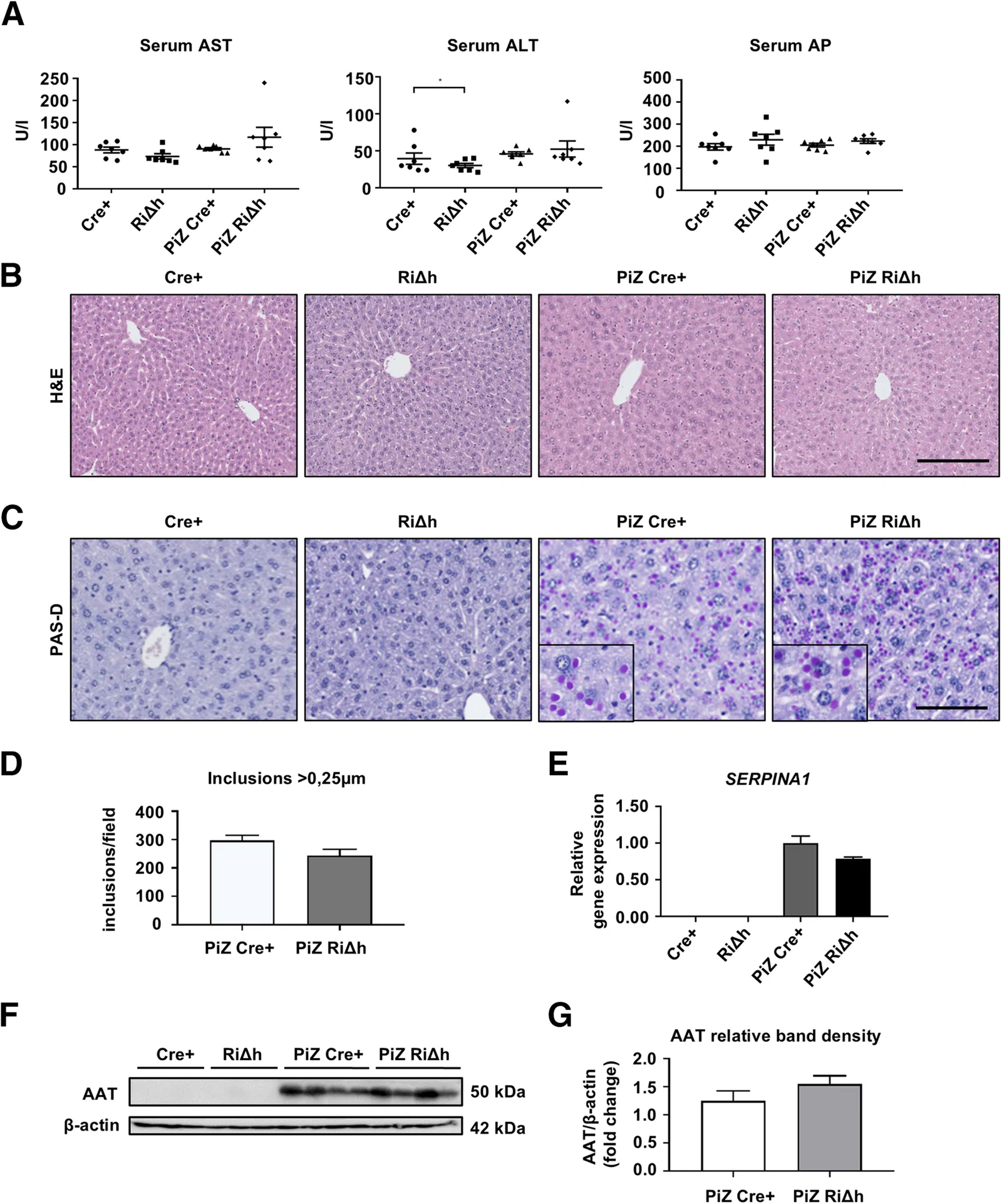
**No obvious alterations in 2-month-old PiZ RictorΔhep mouse livers.** (*A*) Analysis of serum liver enzymes AST, alanine aminotransferase (ALT), and alkaline phosphatase (AP) in 2-month-old Cre+ mice, RICTOR hepatocyte-specific KO mice (RiΔh), PiZ and Cre+ mice (PiZ Cre+), and triple transgenic animals (PiZ RiΔh). (*B*) Representative hematoxylin and eosin (H&E; scale bar, 200 μm) and (*C*) PAS-D staining (scale bar, 100 μm). (*D*) Number of inclusions with a size bigger than 0.25 μm counted with ImageJ. (*E*) qRT-PCR-based relative quantification of *SERPINA1* (AAT gene) levels, compared with ribosomal protein L7 (L7) as an internal control. Messenger RNA levels in PiZ Cre+ were arbitrarily set as 1, and the levels in other groups are presented as ratios. Immunoblot of total liver lysates with an antibody against AAT and β-actin as a loading control with the corresponding morphometric quantification (*F* and *G*). Results are shown as mean ± standard error of the mean (*A*, n = 7; *B–E*, n = 5; *F* and *G*, n = 3–5). Asterisks indicate ∗*P* ≤ .05.

**Figure 4:**
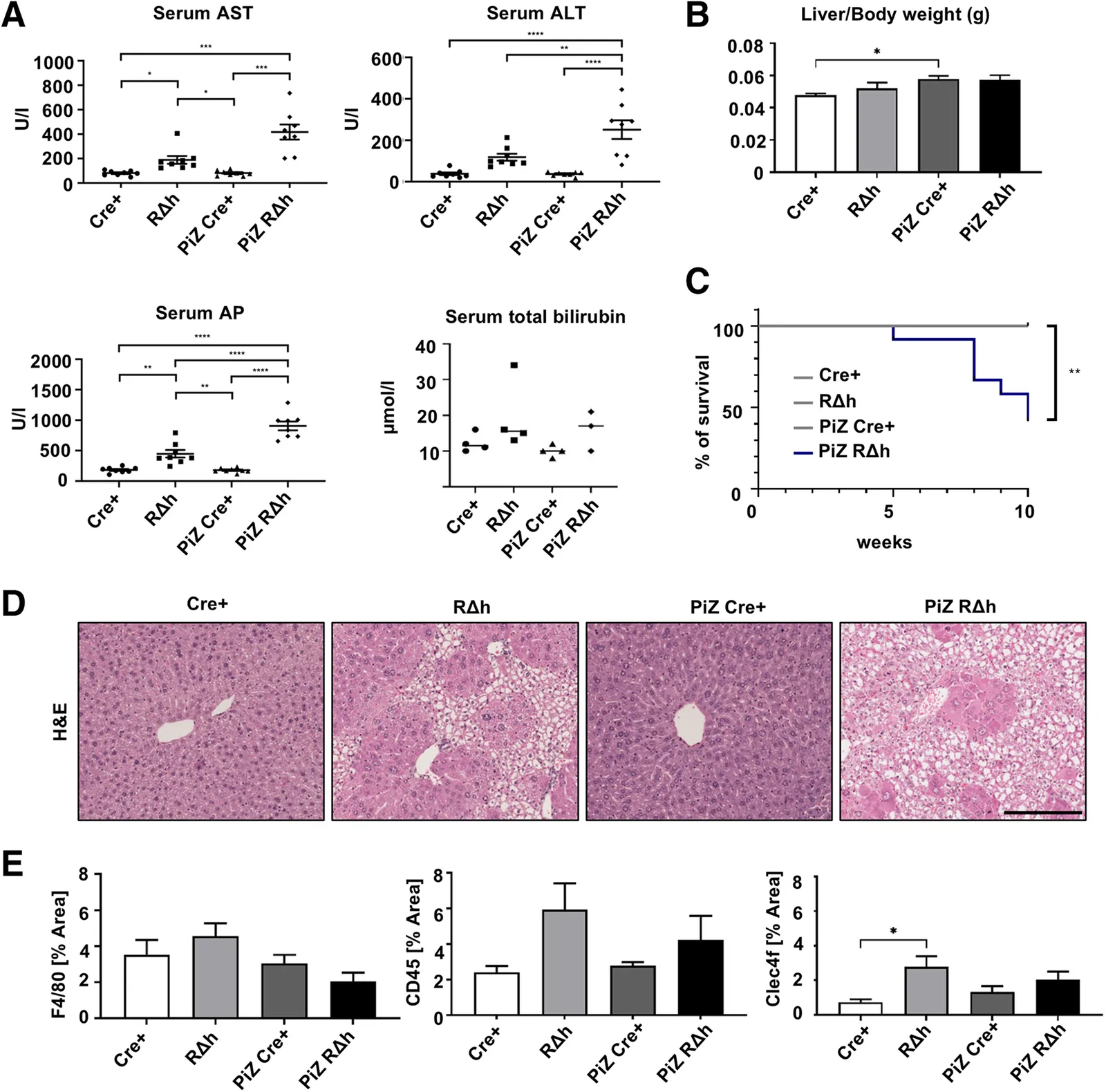
**Increased lethality and aggravated liver injury in 2-month-old PiZ RaptorΔhep mice.** (*A*) Liver enzymes AST, alanine aminotransferase (ALT), and alkaline phosphatase (AP) and serum total bilirubin in sera of 2-month-old albumin Cre+ mice, RAPTOR hepatocyte-specific KO mice (RΔhep), PiZ and Cre+ mice (PiZ Cre+), and triple transgenic animals (PiZ RΔhep). (*B*) Liver-to-body weight ratio. (*C*) Survival of all groups was analyzed up to 10 weeks. (*D*) Representative hematoxylin and eosin (H&E; scale bar, 200 μm) staining. (*E*) Quantification of inflammatory markers, including F4/80 by immunofluorescence and CD45 and Clec4f by immunohistochemistry (n = 12). Results are shown as mean ± standard error of the mean (*A*, n = 5–8; *B*, n = 12; *C*, n = 10; *D*, n = 5; *E*, n = 5–6). Asterisks indicate ∗*P* ≤ .05; ∗∗*P* ≤ .01; ∗∗∗*P* ≤ .001.

**Figure 5:**
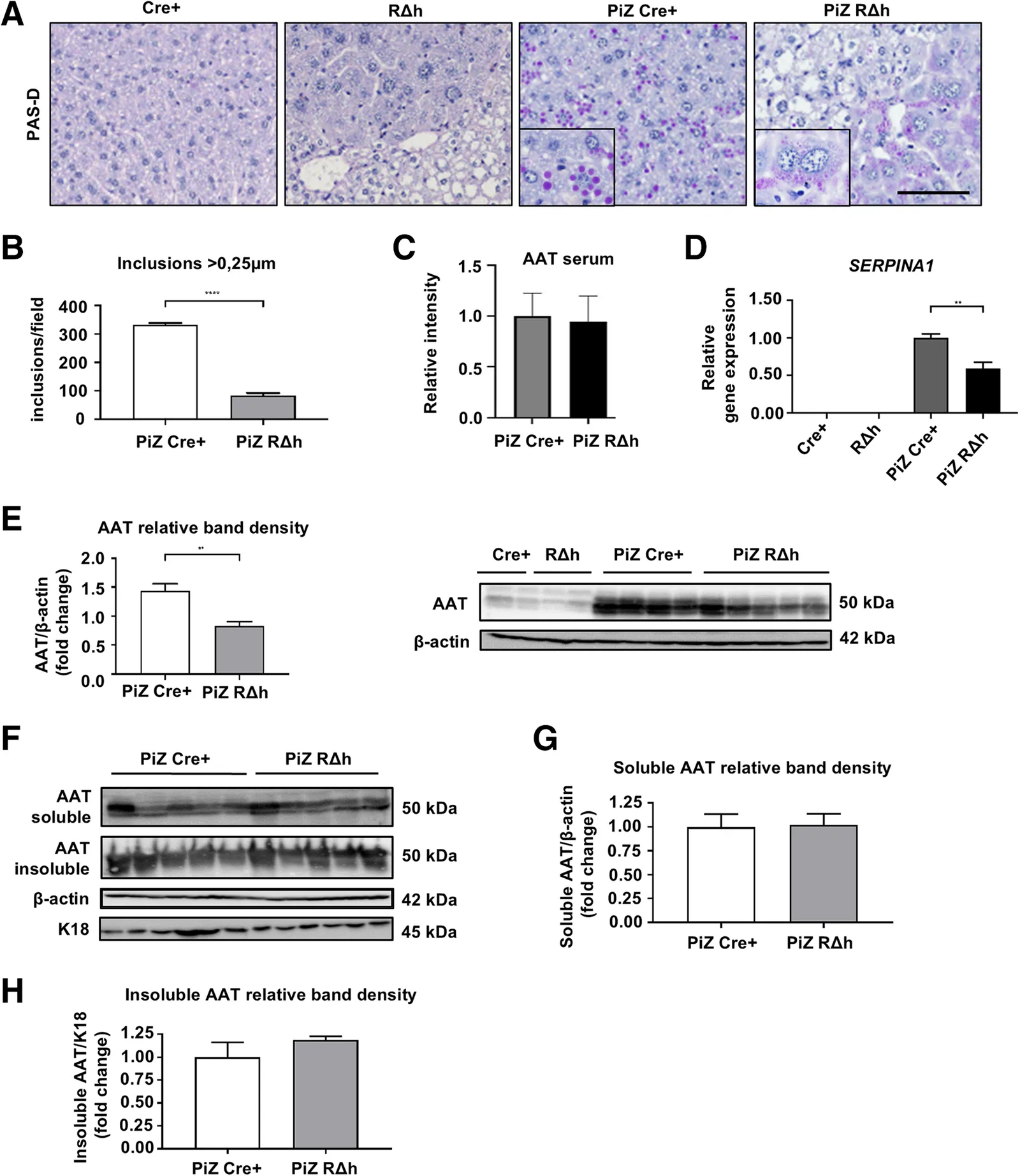
**Reduced AAT levels without changes in soluble or insoluble AAT in 2-month-old PiZ Raptor^Δhep^ mice.** (*A*) Representative PAS-D staining (scale bar, 100 μm). (*B*) Quantification of inclusions with a size bigger than 0.25 μm with ImageJ. (*C*) Dot blot analysis of serum AAT levels. (*D*) qRT-PCR–based relative quantification of *SERPINA1* (AAT gene) levels, with ribosomal protein L7 (L7) used as an internal control. Messenger RNA levels in PiZ Cre+ were arbitrarily set as 1, and the levels in other groups are presented as ratios. Results are shown as mean ± standard error of the mean (n = 5). (*E*) Immunoblot of whole liver lysates with an antibody against AAT, β-actin as a loading control, and the corresponding morphometric quantification. (*F*) Immunoblot from soluble and insoluble liver lysates from 2-month-old mice. Antibodies against AAT, β-actin, and keratin 18 (K18) as a loading control were used, and the corresponding morphometric quantifications are displayed (*G* and *H*). Results are shown as mean ± standard error of the mean. (*A* and *B*, n = 5; *C–E*, n = 3–5; *F–H*, n = 5). Asterisks indicate ∗*P* ≤ .05; ∗∗*P* ≤ .01; ∗∗∗*P* ≤ .001.

**Figure 6:**
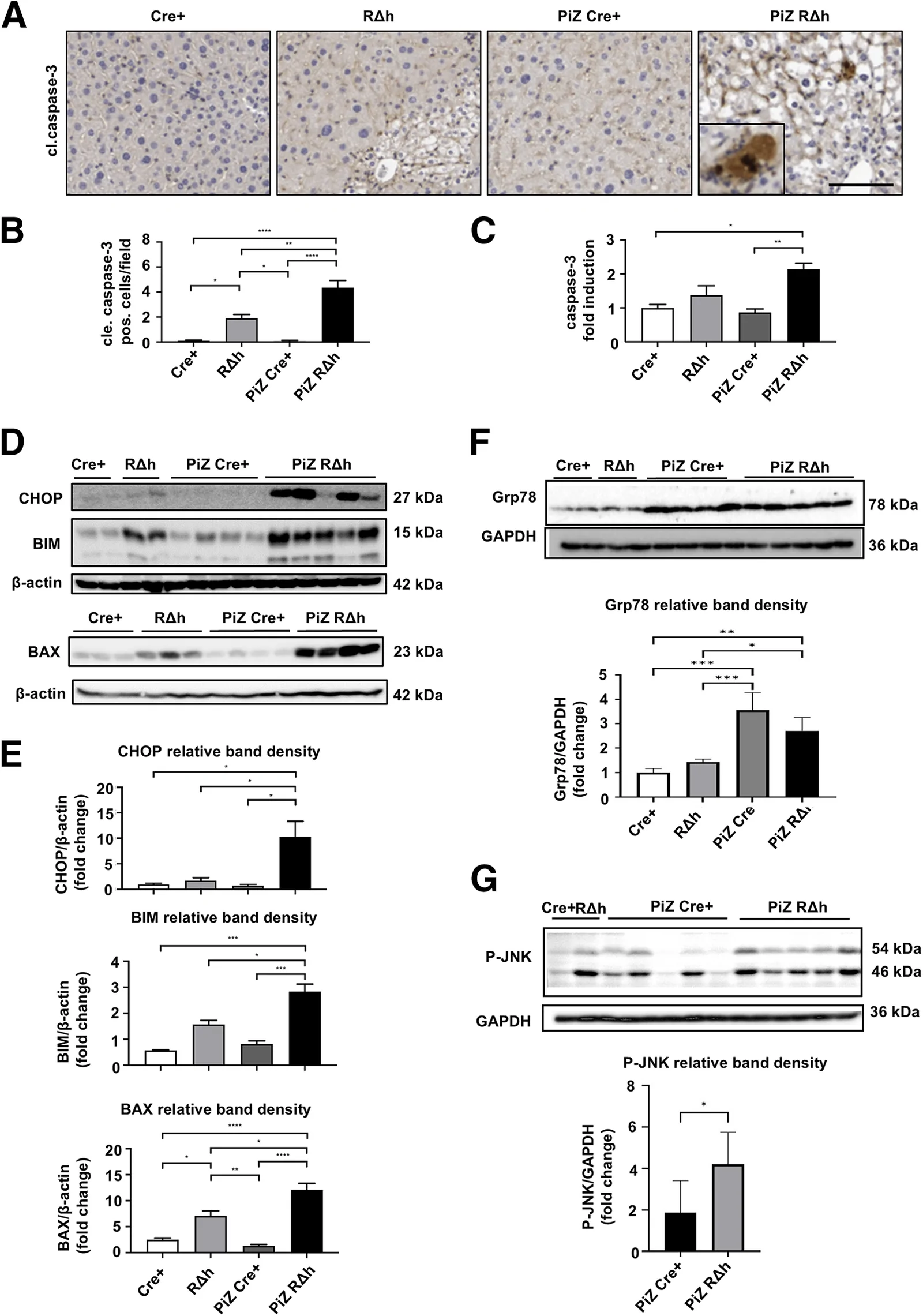
**Increased proapoptotic signaling in 2-month-old PiZ Raptor^Δhep^ mice.** Representative immunohistochemical staining for cleaved caspase-3 (*A*) in livers from 2-month-old Cre+ mice, RAPTOR hepatocyte-specific KO mice (RΔhep), PiZ and Cre+ mice (PiZ Cre+), and triple transgenic animals (PiZ RΔhep). Scale bar, 100 μm. Quantification of caspase-positive cells was done with ImageJ (*B*). (*C*) Caspase-3 assay with Ac-DEVD-AFC caspase-3 fluorogenic substrate. Turnover rate in Cre+ mice was arbitrarily set as 1, and the levels in other groups are presented as ratios. Immunoblot for hepatic CHOP, its downstream targets Bcl-2-like protein 11 (BIM)/Bcl-2-like protein 4 (BAX), and β-actin as a loading control with the corresponding morphometric quantifications (*D* and *E*). Immunoblot for hepatic GRP78 and p-c-Jun N-terminal kinase (JNK) with quantification (*F* and *G*). Results are shown as mean ± standard error of the mean (*A* and *B*, n = 6–7; *C*, n = 5; *D–G*, n = 3–5; *G* and *H*, n = 3–4). Asterisks indicate ∗*P* ≤ .05; ∗∗*P* ≤ .01; ∗∗∗*P* ≤ .001. GAPDH, glyceraldehyde 3-phosphate dehydrogenase.

To determine which 1 of the 2 mTORCs is responsible for the observed phenotype, PiZ animals were mated with either Rictor^Δhep^ and Raptor^Δhep^ animals targeting mTORC2 and mTORC1, respectively. Two-month-old PiZ-Rictor^Δhep^ mice did not display significant differences in liver injury markers AST, alanine aminotransferase, and alkaline phosphatase (Figure 3*A*), liver pathology (Figure 3*B*), number of PAS-D-positive globules, *SERPINA1* expression, and AAT production/accumulation (Figure 3*C–G*). In contrast, PiZ-Raptor^Δhep^ mice exhibited increased serum liver enzymes (Figure 4*A*), whereas total bilirubin levels and liver-to-body weight ratios remained unchanged between PiZ and PiZ-Raptor^Δhep^ mice (Figure 4*A* and *B*). PiZ-Raptor^Δhep^ animals showed a 60% mortality at 10 weeks of age, whereas no deaths were observed in mice with other genotypes (Figure 4*C*). Notably, the mortality was higher in female mice, whereas liver function tests were more elevated in male mice, which might be due to a survival bias (data not shown). Histologic analysis revealed altered liver pathology characterized by hepatocellular ballooning, enlarged hepatocellular nuclei, and minor cholestasis, with only minimal inflammatory changes (Figure 4*D*). Comparable, but much less pronounced, pathology changes were seen in Raptor^Δhep^ animals (Figure 4*D*). Consistently, quantification of inflammatory markers, including F4/80 (macrophages), CD45 (leukocytes), and Clec4f (Kupffer cells), did not show significant differences between groups (Figure 4*E*). Despite the increase mortality, PiZ Raptor^Δhep^ livers displayed a marked reduction in large PAS-D–positive AAT globules (Figure 5*A* and *B*), but serum AAT levels remained unchanged (Figure 5*C*). In contrast, PiZ-Raptor^Δhep^ livers harbored decreased *SERPINA1* messenger RNA and AAT protein levels (Figure 5*D* and *E*). Soluble and insoluble AAT protein levels did not show obvious differences (Figure 5*F–H*). Similar to PiZ-mTOR^Δhep^ animals, PiZ-Raptor^Δhep^ livers had elevated markers of apoptotic cell death (Figure 6*A–C*).

### C/EBP Homologous Protein–Knockout Does Not Rescue the PiZ Raptor^Δhep^ Phenotype

**Figure 7:**
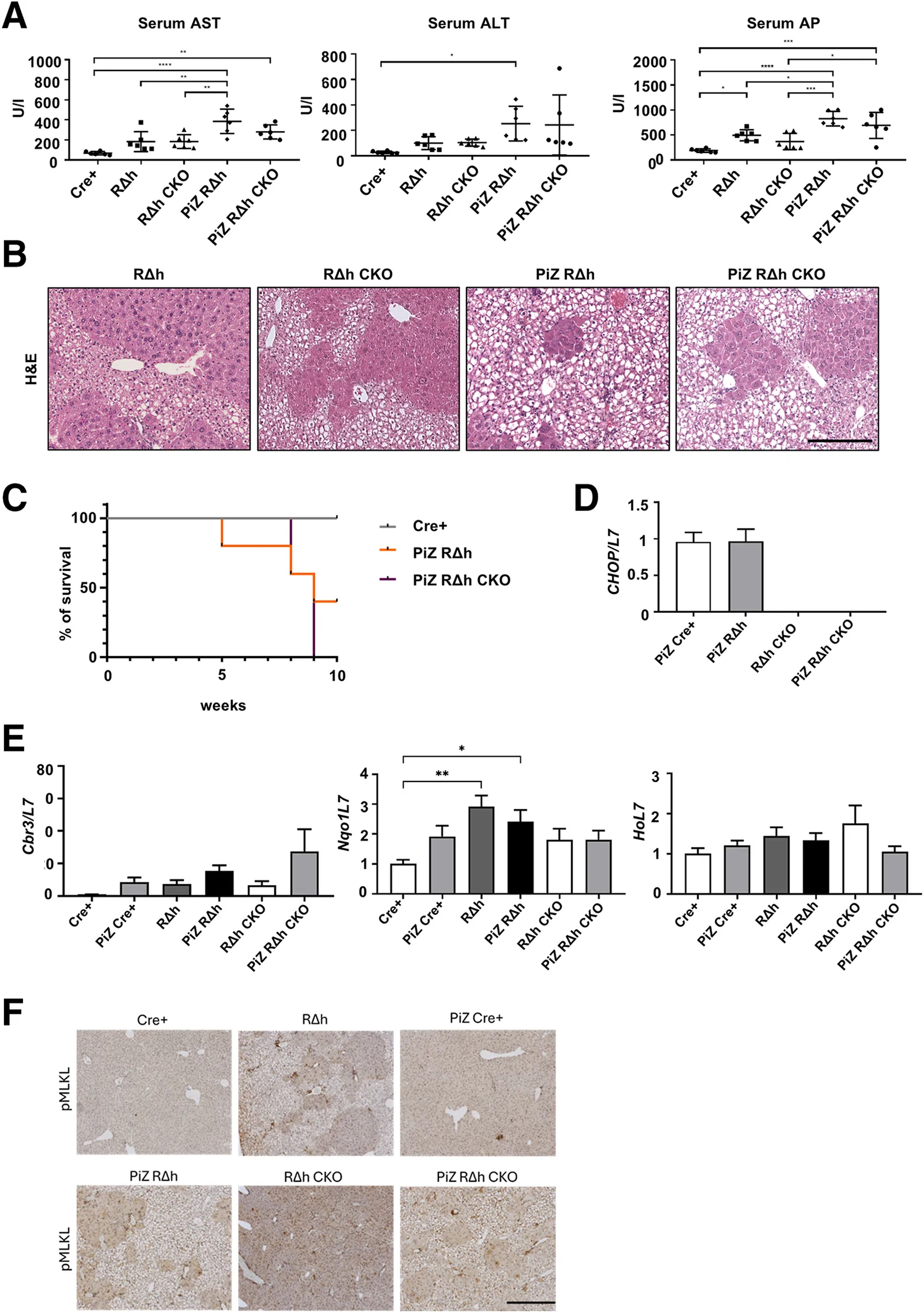
**CHOP knockout does not rescue PiZ Raptor^Δhep^ phenotype.** (*A*) Liver enzymes AST, alanine aminotransferase (ALT), and alkaline phosphatase (AP) of 6- to 7-week old Cre+ mice, RAPTOR hepatocyte-specific KO mice (RΔh), triple transgenic RaptorΔhep mice with constitutive CHOP KO (RΔh CKO), triple transgenic PiZ animals (PiZ RΔh), and quadruple transgenic animals (PiZ RΔh CKO). Results are shown as mean ± standard error of the mean (n = 6). (*B*) Representative hematoxylin and eosin (H&E) staining of liver tissue from 6- to 7-week-old animals. Scale bar, 200 μm. (*C*) Survival was analyzed for 10 weeks with n = 5. (*D*) RT-qPCR analysis of CHOP expression; the levels were normalized to housekeeping gene (ribosomal protein L7 [L7]) and are presented as mean ± standard error of the mean (n = 4–6). In CHOP-KO animals, expression was set to 0 due to undetermined Ct values. (*E*) RT-qPCR analysis of ferroptosis-related markers Cbr3, Ho-1, and Nqo1. Expression levels were normalized to housekeeping gene (L7) and are presented as mean ± standard error of the mean (n = 5–6). (*F*) Representative immunohistochemical staining of pMLKL.(n = 5–6). Scale bar, 200 μm. Asterisks indicate ∗*P* ≤ .05, ∗∗*P* ≤ .01.

Enhanced apoptosis in PiZ Raptor^Δhep^ animals was accompanied by increased levels of the proapoptotic transcription factor CHOP (Figure 6*D* and *E*), together with its elevated downstream targets Bcl-2-like protein 11 and Bcl-2-like protein 4 (Figure 6*D* and *E*). Given that CHOP is a downstream effector of ER stress, we additionally assessed canonical ER stress pathways. However, key ER stress markers, such as GRP78 protein (Figure 6*F*) and ATF6 expression levels (data not shown), were not altered between PiZ and PiZ Raptor^Δhep^ mice, whereas c-Jun N-terminal kinase phosphorylation was increased (Figure 6*G*). To assess the functional relevance of CHOP, we crossbred PiZ Raptor^Δhep^ mice with constitutive CHOP-KO animals. However, CHOP ablation did not improve liver enzymes (Figure 7*A*), pathologic liver changes (Figure 7*B*), or mouse survival (Figure 7*C*), suggesting that CHOP is not the key determinant of the observed phenotype. Efficient CHOP deletion was confirmed by quantitative real-time polymerase chain reaction (RT-qPCR) (Figure 7*D*).

To explore alternative cell death pathways, we assessed markers of ferroptosis (Cbr3, Ho-1, Nqo1) by RT-qPCR and necroptosis (pMLKL) by immunohistochemistry. No significant differences were observed between the PiZ-expressing groups (Figure 7*E* and *F*).

### PiZ Raptor^Δhep^ Mice Show Decreased Levels of Key Liver Regeneration Factors but No Signs of a Critical Liver Synthesis Impairment

**Figure 8:**
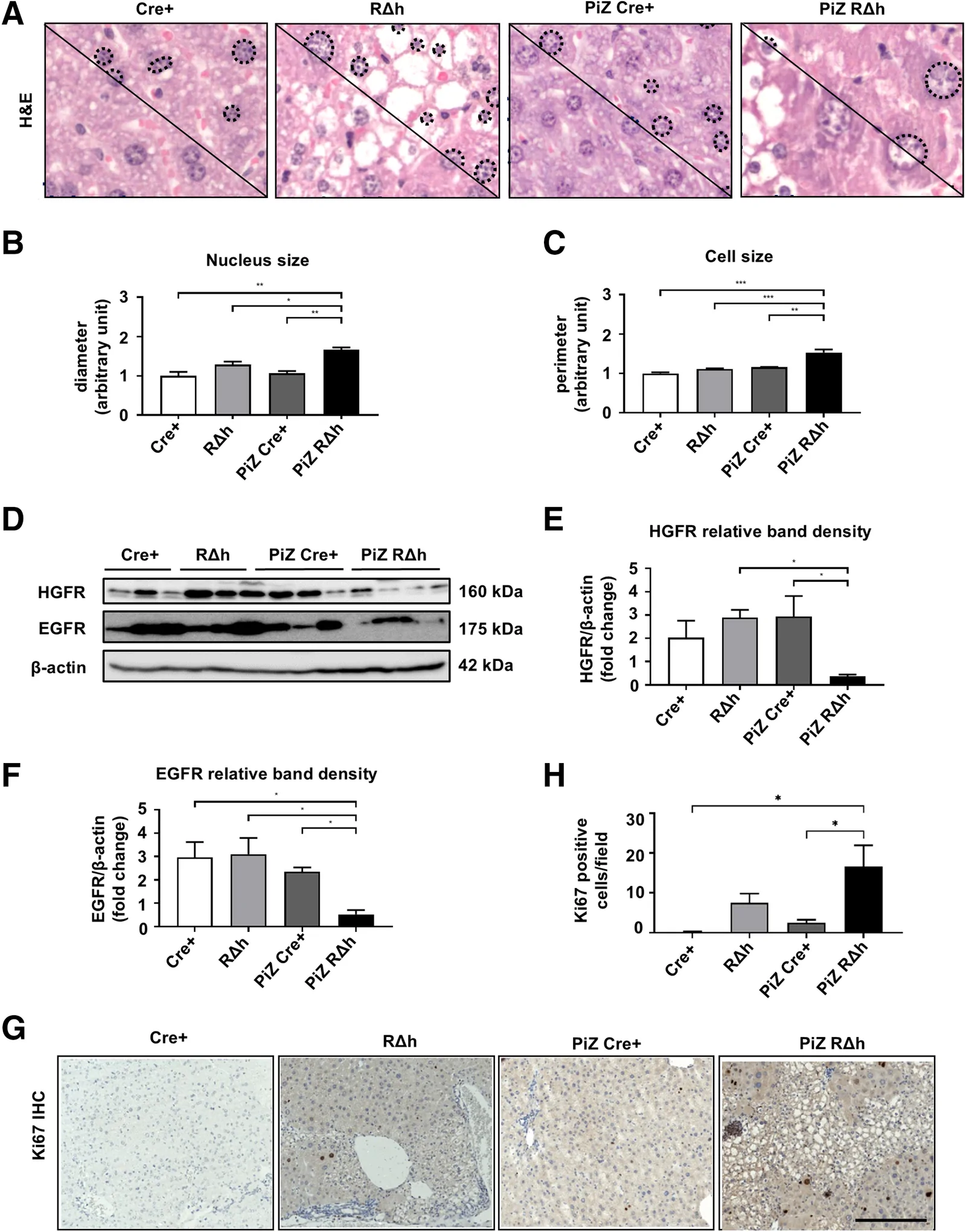
**Altered nuclear morphology and liver regeneration/proliferation pathways in PiZ RaptorΔhep mice.** (*A*) Representative hematoxylin and eosin (H&E) staining from 2-month-old Cre+ mice, RAPTOR hepatocyte-specific KO mice (RΔhep), PiZ and Cre+ mice (PiZ Cre+), and triple transgenic animals (PiZ RΔhep). Hepatocyte nuclei are highlighted with *dashed blue lines*. Scale bar, 50 μm. Nucleus (*B*) and cell diameter (*C*) were measured with QuPath. (*D*) Immunoblotting of whole liver lysates for hepatocyte growth factor receptor (HGFR), epidermal growth factor receptor (EGFR), and β-actin as a loading control with the corresponding morphometric quantifications (*E* and *F*). Representative immunohistochemical (IHC) staining for Ki67 on liver sections from 2-month-old mice of the indicated genotypes (*G*). Quantification of Ki67-positive nuclei demonstrates increased hepatocyte proliferation in PiZ RaptorΔhep livers compared with controls (*H*). Results are shown as mean ± standard error of the mean (*A–C*, n = 3; *D–F*, n = 3–4; *G* and *H*, n = 4–5). Asterisks indicate ∗*P* ≤ .05. Scale bar, 200 μm.

**Figure 9:**
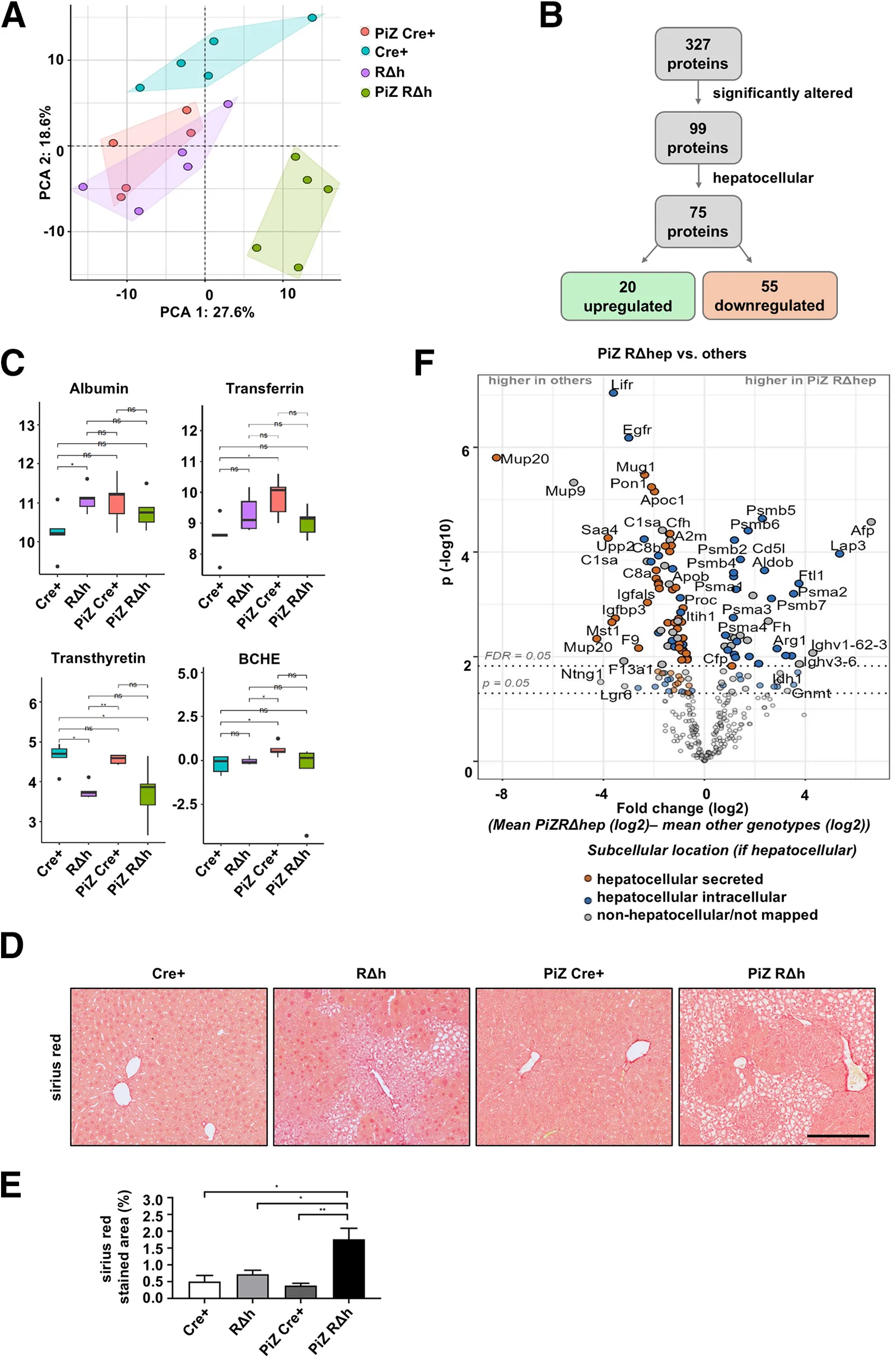
**Altered serum proteome and mild fibrosis in 2-month-old PiZ RaptorΔhep mice.** (*A*) PCA of serum proteome of Cre+ mice (Cre+), RAPTOR hepatocyte-specific KO mice (RΔhep), PiZ and Cre+ mice (PiZ Cre+), and triple transgenic animals (PiZ RΔhep). Five individual mice were studied per group. (*B*) Flow chart depicts the number of reproducibly detected proteins (n = 327) and the number of significantly altered proteins (n = 99) (FDR <0.05). Among those, 75 were of hepatocellular origin (20 upregulated and 55 downregulated in PiZ RΔhep mice compared with other genotypes). (*C*) Intensities of selected hepatocyte-made serum proteins albumin, transferrin, cholinesterase, and apolipoprotein B are displayed. (*D*) Representative Sirius red staining and the corresponding quantification (*E*) of livers from 2-month-old Cre+ mice (Cre+), RAPTOR hepatocyte-specific KO mice (RΔhep), PiZ and Cre+ mice (PiZ Cre+), and triple transgenic animals (PiZ RΔhep). Scale bar, 200 μm. (*F*) Volcano plot illustrates differentially abundant proteins from serum proteomics when comparing triple transgenic animals (PiZ RΔhep) with all other groups. The −log10 (*P* value) is plotted on the y-axis against the log2-fold change (revealing the differences between means of log2-transformed label-free quantification intensities) on the x-axis. A log2-fold change >0 indicates proteins elevated in PiZ RΔhep mice, whereas a value <0 identifies diminished features. *Dashed lines* indicate significance thresholds (*P* < .05 or FDR-adjusted *P* value < .05). Proteins of hepatocellular origin were classified by subcellular location based on their UniProt annotation and are highlighted among significantly altered proteins (*P* < .05). Results are shown as mean ± standard error of the mean (*A–C,* and *F*, n = 5; *D* and *E*, n = 3). ∗*P* ≤ .05, ∗∗*P* ≤ .01, and ns = *P* ≥ .05.

Because PiZ Raptor^Δhep^ animals displayed increased nuclei and cell size (Figure 8*A–C*), we analyzed the key regeneration pathways and detected decreased levels of epidermal growth factor receptor and hepatocellular growth factor receptor (Figure 8*D–F*). Consistent with largely unchanged liver mass, hepatocyte proliferation, as assessed by Ki67 staining, was not impaired and was even somewhat elevated in PiZ Raptor^Δhep^ livers (Figure 8*G*). To test whether the decreased survival of PiZ Raptor^Δhep^ animals is due to a failing liver synthesis function, serum proteomics was performed. In the principal component analysis (PCA), sera of PiZ Raptor^Δhep^ animals clearly differed from the remaining groups (Figure 9*A*). Of 327 identified proteins, 99 differed significantly (false discovery rate [FDR] <0.05) between PiZ Raptor^Δhep^ and all other groups. Of them, 75 were of hepatocellular origin, 55 being downregulated and 20 being upregulated in PiZ Raptor^Δhep^ animals (Figure 9*B* and Supplementary Table 1). Collectively, although these data suggest markedly altered hepatocellular protein synthesis, the changes were much less pronounced than alterations observed in human liver cirrhosis.^32^ In particular, the amounts of characteristic hepatocyte synthesis markers (albumin, transferrin, butyrylcholinesterase, and transthyretin) were unaltered (Figure 9*C*). In line with this preserved hepatocellular function, only minor fibrosis and no signs of portal hypertension were seen (Figure 9*D* and *E*). Serum proteomics are summarized in a Volcano plot revealing diminished levels of various secreted hepatocellular products, whereas hepatocellular injury markers were mostly elevated (Figure 9*F*; Supplementary Table 1). Increased alpha-fetoprotein and decreased leukemia inhibitory factor receptor serum levels pointed to an increased abundance of immature hepatocytes in PiZ Raptor^Δhep^ livers (Figure 9*F*).

### Hepatocellular Regulatory-Associated Protein of Mammalian Target of Rapamycin Knockout Leads to Metabolic Alterations in PiZ Mice

**Figure 10:**
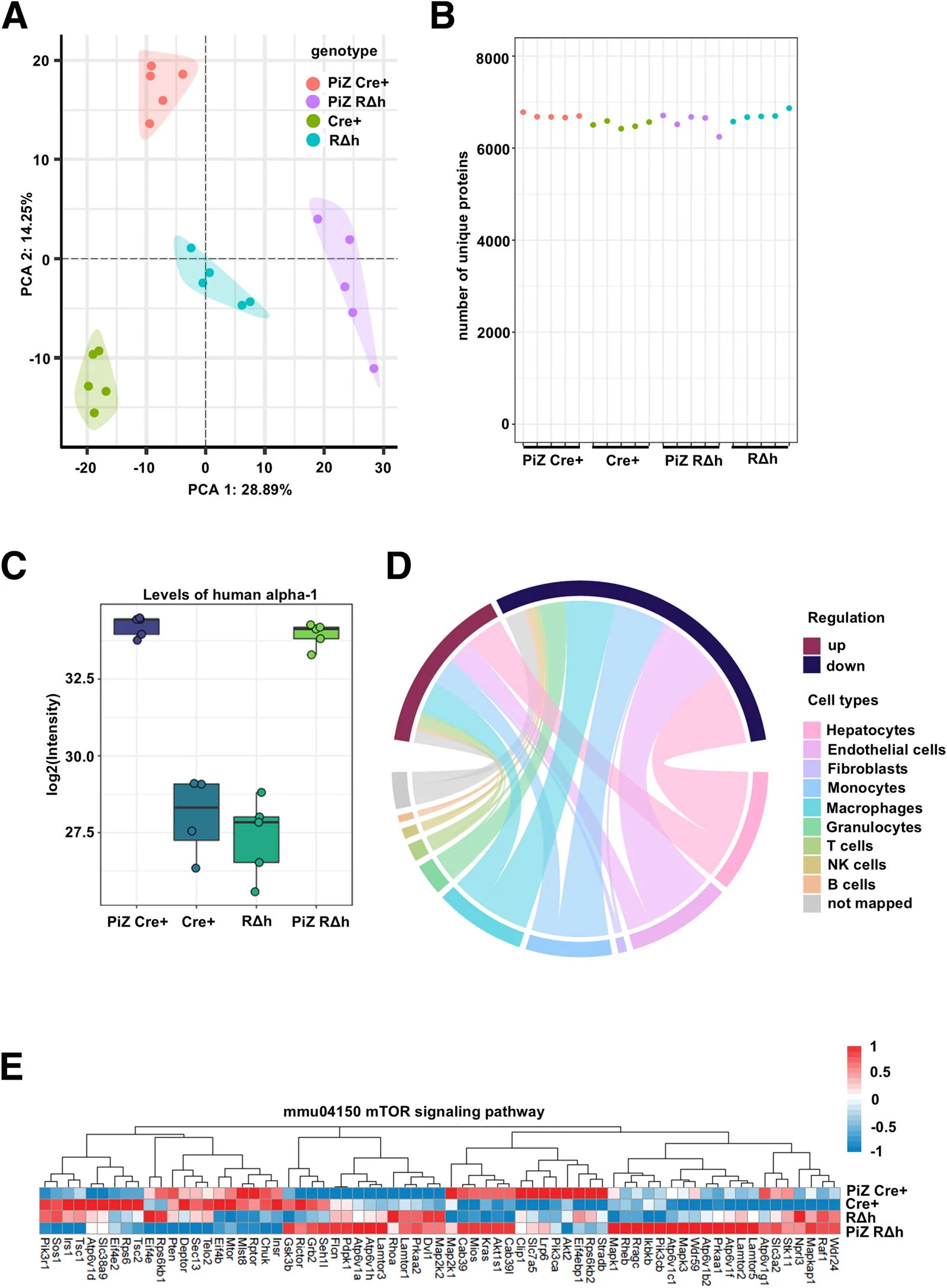
**Bulk liver proteomics of 2-month-old animals.** (*A*) PCA of bulk liver proteomics of Cre+ mice (Cre+), RAPTOR hepatocyte-specific KO mice (RΔhep), PiZ and Cre+ mice (PiZ Cre+), and triple transgenic animals (PiZ RΔhep). (*B*) Number of unique proteins per sample. (*C*) Levels of human AAT (UniProt ID, P01009). (*D*) Chord plot depicts the number of proteins significantly (FDR <0.05) increased and decreased in livers of triple transgenic animals (PiZ RΔhep) compared with mice expressing PiZ and Cre+ (PiZ Cre+) subdivided based on their cellular origin. (*E*) Levels of individual proteins assigned to the Kyoto Encyclopedia of Genes and Genomes (KEGG) pathway ‘mTOR signaling’ (mmu04150) color-coded based on their z scores (*A–E*, n = 5).

**Figure 11:**
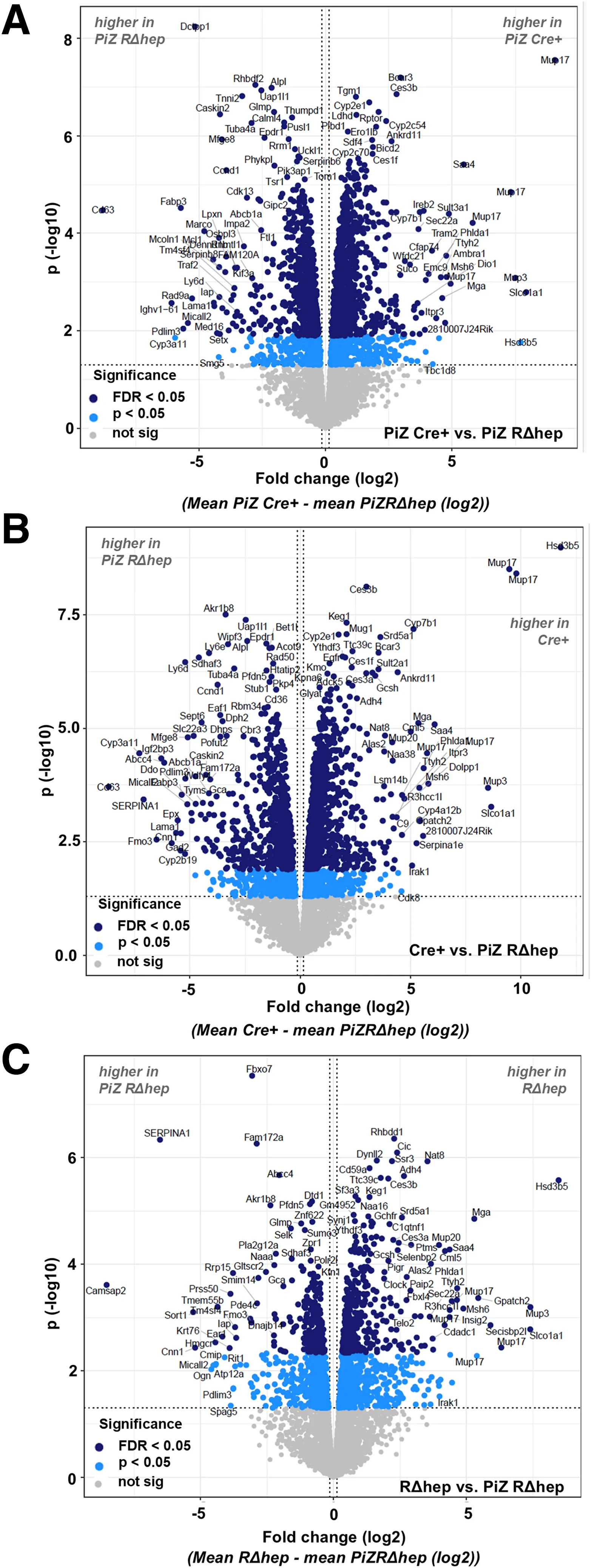
**Proteomic alterations in PiZ RaptorΔhep animals. (***A–C*) Volcano plots illustrates differentially abundant proteins in bulk liver proteomics when comparing Cre+ mice (Cre+) or Cre+ mice with PiZ expression (PiZ Cre+) and triple transgenic animals (PiZ RΔhep) (*A* and *B*) or RAPTOR hepatocyte-specific KO mice (RΔhep) and triple transgenic animals (PiZ RΔhep, *C*). The x- and y-axes indicate the fold change and the *P* value, respectively. Significantly altered proteins with a FDR <0.05 are labeled in *dark blue*, and proteins with a *P* value < .05 in *light blue*. *Gray color* indicates not significantly altered proteins. The −log10 (FDR-adjusted *P* value) is plotted on the y-axis against the log2-fold change on the x-axis, depicting the differences between means of log2-transformed label-free quantification intensities. A log2-fold change <0 indicates proteins upregulated in PiZ RΔhep mice, whereas a value >0 identifies downregulated species. Statistically significant proteins with a FDR <0.05 are labeled in *dark blue*, and proteins with a *P* value < .05 are labeled in *light blue*. *Gray color* indicates not significantly altered proteins.

**Figure 12:**
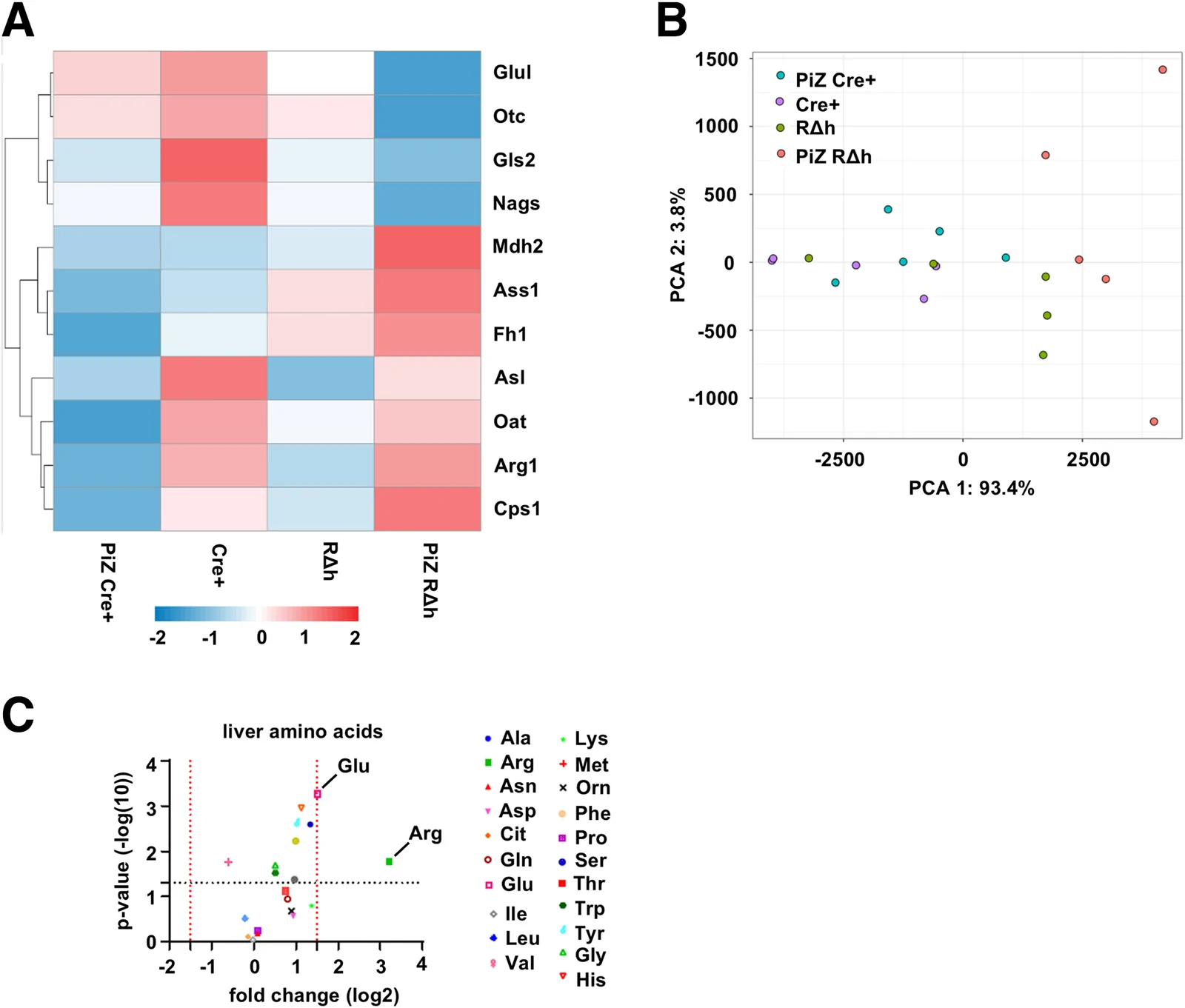
**Metabolic changes and altered zonation in PiZ RaptorΔhep livers.** (*A*) Color-coded z scores of selected key proteins of the urea cycle are shown. Cre+, albumin Cre recombinase expressing mice; RΔh, RAPTOR hepatocyte-specific KO mice. (*B*) PCA of liver small metabolite measurements in Cre+ mice (Cre+), RAPTOR hepatocyte-specific KO mice (RΔhep), PiZ and Cre+ mice (PiZ Cre+), and triple transgenic animals (PiZ RΔhep) (n = 4). (*C*) Hepatic levels of highlighted small metabolites increased (>1) and decreased (<1) in PiZ RΔhep vs PiZ Cre+ animals. Five individual mice per group were analyzed.

To understand why PiZ Raptor^Δhep^ livers showed signs of decreased differentiation and to clarify the mechanisms leading to PiZ Raptor^Δhep^ liver injury, a proteomic analysis of total liver lysates was performed and showed distinct clustering of the 4 transgenic mouse lines (Figure 10*A*). Quality control (QC) revealed a comparable number of unique proteins in all groups as well as increased levels of human AAT in PiZ mice harboring Cre recombinase (with albumin promotor) (Cre+) and PiZ Raptor^Δhep^ animals (Figure 10*B* and *C*). Compared with all control groups, 519 proteins were upregulated, and 992 were downregulated in PiZ Raptor^Δhep^ animals. Notably, proteins mapped to inflammatory cells were mostly decreased. Given the severe phenotype of the PiZ Raptor^Δhep^ mice, the lack of inflammation points towards an overall downregulated liver function (Figure 10*D*). Kyoto Encyclopedia of Genes and Genomes pathway analysis confirmed the expected alterations in mTOR-related proteins in Raptor^Δhep^ and PiZ Raptor^Δhep^ animals (Figure 10*E*). Bulk liver proteomics also revealed changes in proteins that are involved in metabolic functions (Figure 11*A–C*), such as urea cycle–associated proteins (Figure 12*A*). To further investigate these metabolic changes, an assessment of key liver metabolites was performed. PCA showed relatively large interindividual differences with PiZ Raptor^Δhep^ animals differing from the remaining groups (Figure12B). Several amino acids and small metabolites were altered in livers of PiZ Raptor^Δhep^ animals (Supplementary Table 2), with glutamic acid and arginine being particularly increased in PiZ Raptor^Δhep^ livers compared with Cre+ animals (Figure 12*C*).

### Disturbed Liver Zonation Leads to Toxic Hyperammonemia in PiZ Raptor^Δhep^ Mice Livers

**Figure 13:**
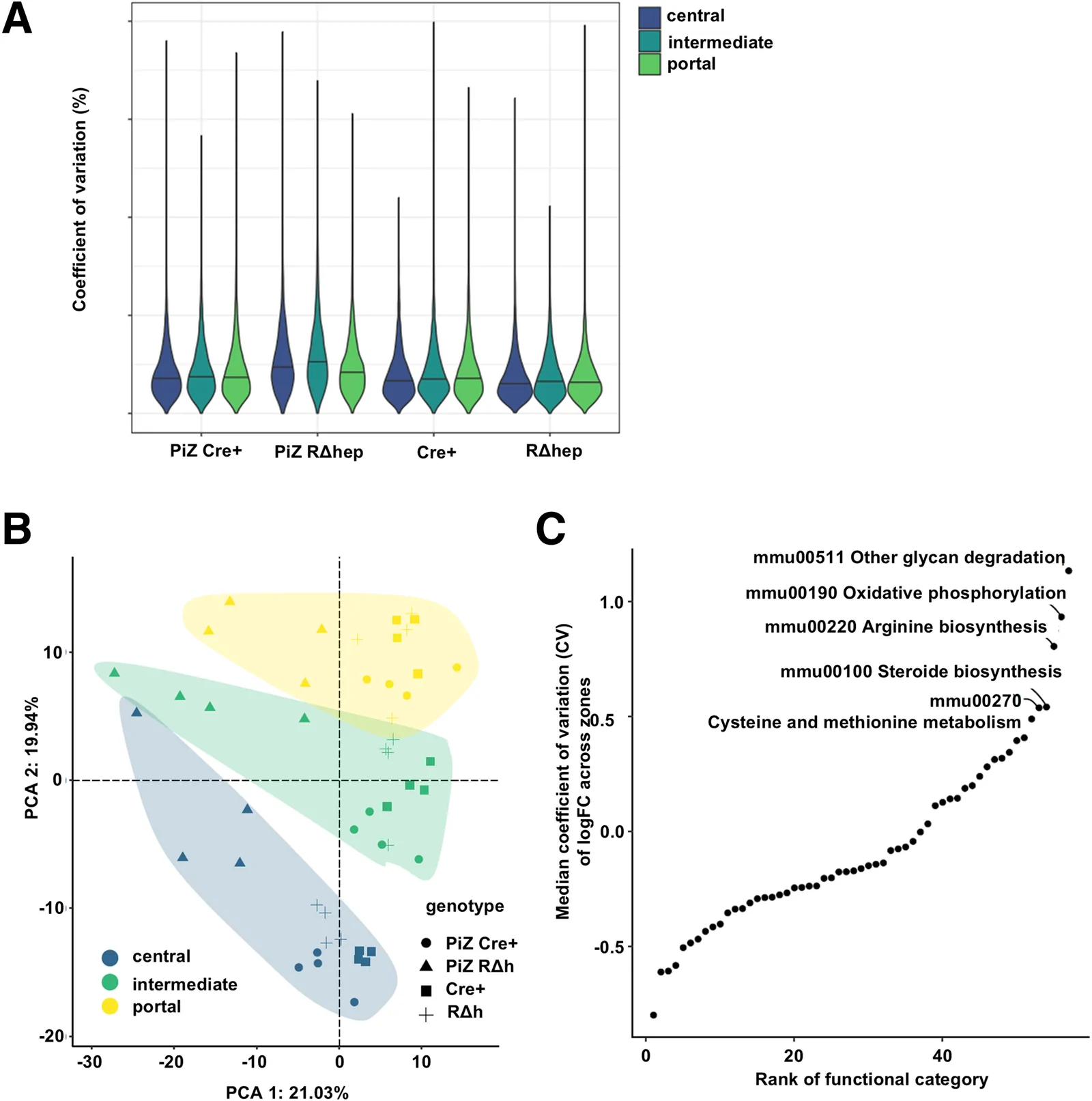
**Disturbed liver zonation and hyperammonemia in PiZ RaptorΔhep mice.** (*A*) Coefficient of variation of spatial liver proteomics from 2-month-old animals. (*B*) PCA of spatial liver proteomics. (*C*) Functional protein groups that differ significantly across the 3 assessed hepatic zones (gene set enrichment analysis; FDR < 0.1) were ranked by calculating the median coefficient of variation (CV) of fold changes for each protein in the respective pathway along the zonation axis. The top 5 Kyoto Encyclopedia of Genes and Genomes groups are highlighted. PiZ RΔhep animals are compared with all other groups. (*A* and *B*, n = 5; *C*, n = 4).

**Figure 14:**
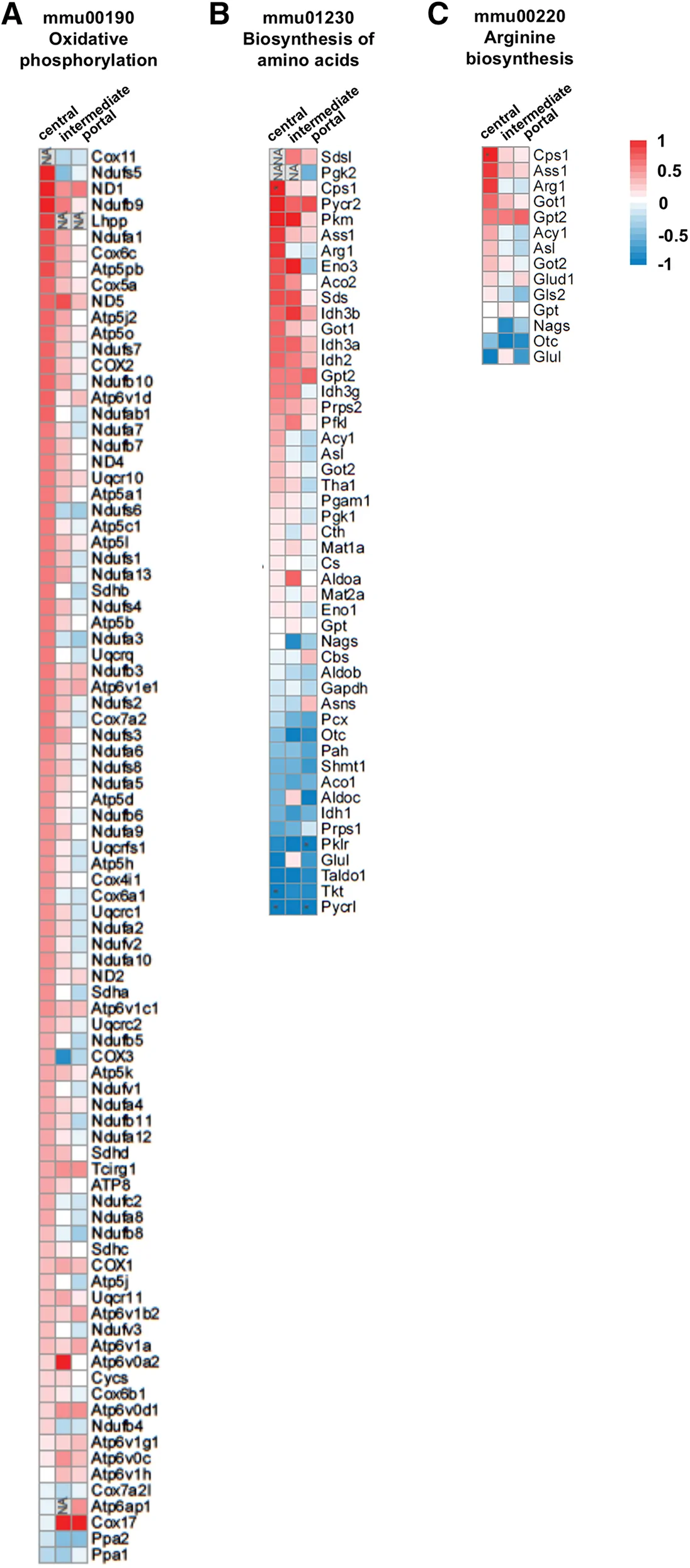
**Overview of selected Kyoto Encyclopedia of Genes and Genomes (KEGG) pathways assessed in spatial liver proteomics.** Overview of proteins assigned to KEGG pathways oxidative phosphorylation (mmu00190) (*A*), amino acids biosynthesis (mmu01230) (*B*), and arginine metabolism (mmu0020) (*C*). PiZ RΔhep triple transgenic animals compared with all other groups. Colors indicate z scores per protein in portal, intermediate, and central area.

**Figure 15:**
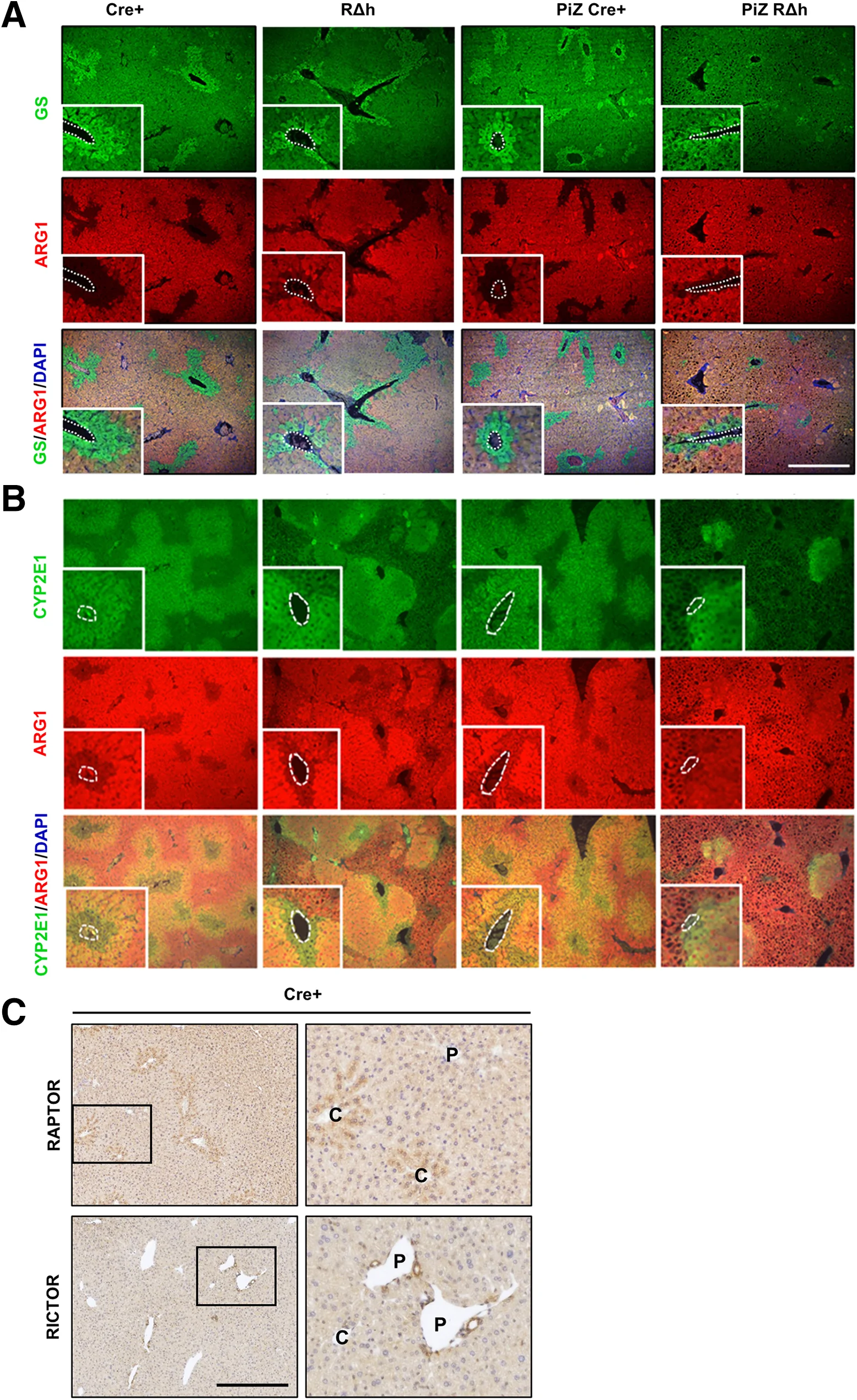
**Zonal distribution and levels of selected proteins.** (*A* and *B*) Immunofluorescent staining for the pericentral markers glutamine synthetase (GS, *green*) and cytochrome P450 2E1 (CYP2E1; *green*) in combination with arginase-1 (ARG-1; *red*) in livers from 2-month-old Cre+ mice (Cre+), Raptor hepatocyte-specific KO mice (RΔh), PiZ and Cre+ mice (PiZ Cre+), and triple transgenic animals (PiZ RΔh). Nuclei are stained *blue* with 4′,6-diamidino-2-phenylindole (DAPI). (*C*) Representative immunohistological staining for RAPTOR and RICTOR on liver samples from 2-month-old Cre+ mice. Scale bars, 200 μm and magnification of the indicated area. C, central vein; P, portal triad. (*A–C*, n = 5).

**Figure 16:**
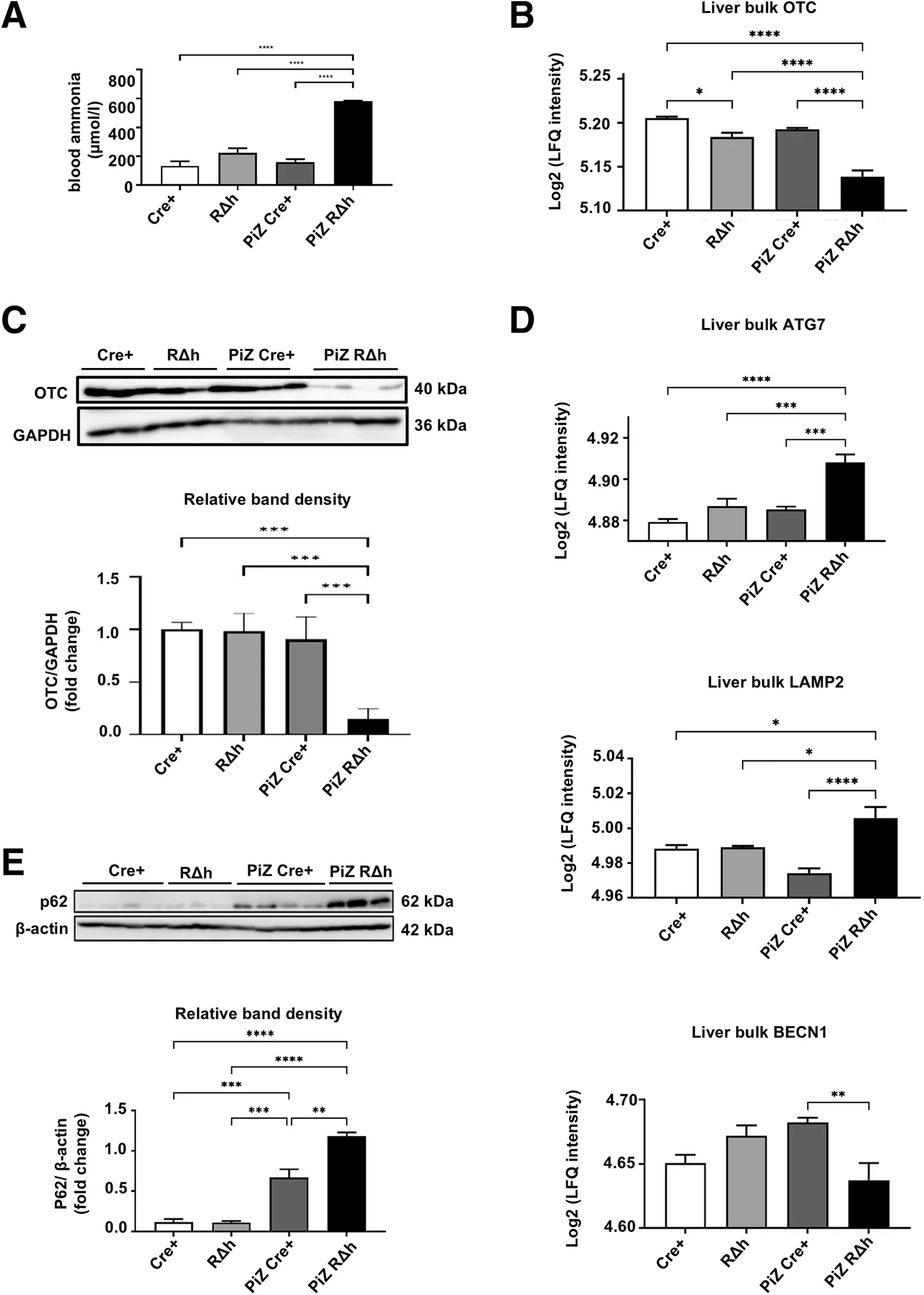
**Hyperammonemia and altered hepatic levels of autophagy-related proteins in PiZ RaptorΔhep mice.** (*A*) Blood ammonia levels were measured with the DRI-CHEM NX10N blood ammonia analyzer. (*B*) Label-free quantification (LFQ)-based quantification of ornithine transcarbamylase (OTC) derived from bulk proteomics analysis. (*C*) Immunoblotting visualizes OTC and glyceraldehyde-3-phosphate dehydrogenase (GAPDH) as a loading control with the corresponding morphometric quantifications. (*D*) LFQ-based quantification of selected proteins (ATG7, LAMP2, and BECLIN1) derived from liver bulk proteomics analysis. Data are presented as log2 (LFQ intensity) with mean ± standard error of the mean. (*E*) Immunoblot analysis of hepatic p62 with β-actin as a loading control (*A, B,* and *D*, n = 5; *C* and *E*, n = 3–4). Asterisks indicate ∗*P* ≤ .05; ∗∗*P* ≤ .01; ∗∗∗*P* ≤ .001; ∗∗∗∗*P* ≤ .0001.

Collectively, both bulk proteomics and metabolite profiling demonstrated a profound alteration of hepatocellular function, with changes in liver zonation–associated processes such as glutamic acid metabolism. To further characterize them, we generated a spatially resolved liver proteome by Deep Visual Proteomics.^33^ The workflow comprised staining, imaging, artificial intelligence–guided selection of 3 zones (periportal, intermediary, pericentral), laser microdissection, and high-sensitivity mass spectrometry. Coefficients of variation per protein were at 22.7% (median) and were lowest for PiZ Raptor^Δhep^ samples, indicating a robust peptide and liquid chromatography–mass spectrometry workflow (Figure 13*A*). PCA analysis revealed a marked loss of zonation in PiZ Raptor^Δhep^ hepatocytes, in that the cells from all 3 liver zones were much more similar than the hepatocyte subtypes from other transgenic lines. The changes were particularly strong in pericentral PiZ Raptor^Δhep^ hepatocytes (Figure 13*B*). Oxidative phosphorylation, arginine biosynthesis, and steroid biosynthesis, 3 highly zonation-dependent processes, were among the top 5 most altered functional categories, further substantiating an altered liver zonation in PiZ Raptor^Δhep^ animals, confirming the findings from the metabolite analysis (Figure 13*C*; Figure 14*A–C*). Immunofluorescence staining for glutamine synthetase (GS) as a pericentral hepatocyte marker and arginase-1 (ARG-1) as a periportal/intermediate hepatocyte marker confirmed a diminished number of GS-positive cells in PiZ Raptor^Δhep^ livers. GS and ARG-1 staining was complementary in all genotypes (ie, ARG-1–positive cells were negative for GS and vice versa) and demonstrates a loss of pericentral cells in PiZ Raptor^Δhep^ livers (Figure 15*A*). Analysis of the pericentral marker cytochrome P450 2E1 further supported this finding (Figure 15*B*). To further substantiate these findings, staining of Cre+ mice confirmed the predominantly pericentral abundance of RAPTOR, whereas RICTOR staining was strongest in the periportal area (Figure 15*C*). The marked changes seen in pericentral hepatocytes of PiZ Raptor^Δhep^ animals, together with increased glutamic acid levels, prompted us to measure ammonia levels. Blood analysis demonstrated that these were markedly increased and likely led to death of these metabolically reprogrammed mice (Figure 16*A*). Consistent with impaired ammonia detoxification, expression of the urea cycle enzyme ornithine transcarbamylase was diminished in PiZ Raptor^Δhep^ mice, as shown by bulk proteomics (Figure 16*B*) and immunoblotting analysis (Figure 16*C*). Given the observed metabolic alterations and impaired ammonia detoxification, as well as the known link between mTORC1 signaling and autophagy, we examined components/surrogates of the autophagy–lysosomal pathway. Analysis of the liver bulk proteomics dataset revealed reduced BECN1 alongside increased ATG7 and LAMP2 levels in PiZ Raptor^Δhep^ mice (Figure 16*D*). Immunoblotting analysis demonstrated elevated hepatic p62 levels (Figure 16*E*).

## Discussion

Given the importance of mTOR signaling in proteostasis, as well as previous findings indicating the efficacy of mTOR inhibition in AATD-LD,^13^^,^^15^ we systematically explored the relevance of hepatocellular mTOR signaling in an animal model of this disorder. Although PiZ-mTOR^Δhep^ and PiZ-Raptor^Δhep^ mice displayed an unaltered or even decreased AAT accumulation, they both displayed increased liver injury, which is in contrast with multiple previous transgenic mouse studies,^34^ as well as human data demonstrating the key relevance of proteotoxic stress in AATD-LD.^8^^,^^11^^,^^35^ In addition to the ablated mTOR signaling that was previously shown to increase autophagic degradation, an increased hepatocellular injury and associated hepatocyte loss/dedifferentiation may have contributed to the decreased hepatic AAT levels in PiZ-Raptor^Δhep^ mice.^16^, ^17^, ^18^ Further studies are needed to comprehensively evaluate the autophagic machinery and to dissect the contribution of the described processes to the observed phenotype. Despite increased hepatocellular apoptosis, elevated CHOP levels, and the previously demonstrated significance of CHOP in experimental AATD-LD,^31^ CHOP ablation was not able to rescue PiZ-Raptor^Δhep^, suggesting that the underlying pathology is more complex.

A detailed proteomic analysis revealed an altered hepatic zonation as a potential mechanism. Although altered zonation in PiZ mice has been reported previously,^36^ we used a strain with lower PiZ expression that did not display a markedly altered zonation at the baseline, and the same was true for Raptor^Δhep^ animals, despite the fact that nutrient/hormone sensing constitutes an important driver of hepatic zonation.^37^ In contrast, PiZ-Raptor^Δhep^ mice displayed a markedly altered hepatic zonation with multiple metabolic alterations. This is in line with reports demonstrating that chronic liver disease results in altered zonation,^38^ and we therefore assume that the combination of both genetic hits led to the observed phenotype. Although we detected alterations in important regenerative pathways, which is in line with the known importance of mTOR signaling in liver regeneration,^39^^,^^40^ Ki67 staining revealed a somewhat increased, likely compensatory proliferation in PiZ-Raptor^Δhep^ mice. In line with that, we observed neither a decreased liver mass (data not shown) nor altered serum bilirubin levels. Despite somewhat reduced serum levels of multiple hepatocellular-secreted proteins, these findings suggest that the observed fatal phenotype is not due to a secretory liver failure, although a minor contribution cannot be fully excluded.

Metabolic profiling revealed increased hepatic arginine levels in PiZ-Raptor^Δhep^ mice. Z-AAT protein contains <2% of arginine residues, which is relatively low compared with the average frequency of 5% to 6% in eukaryotes.^41^^,^^42^ Therefore, we argue that overexpression may, in combination with the comparable low Z-AAT arginine amount, lead to an overall arginine surplus. Notably, arginine is known to lead to metabolic reprogramming and may therefore contribute to some of the observed changes, as well as a compensatory activation of mTOR signaling that is ablated in these mice.^43^^,^^44^ However, further studies, such as targeted amino acid profiling, a thorough assessment of urea-cycle enzyme activities, and/or isotopic flux using tracers, as well as exclusion of intake or catabolic confounders, are needed to substantiate this hypothesis.

The changes in liver zonation seen in PiZ-Raptor^Δhep^ mice were most pronounced in the pericentral hepatocytes, which is in line with studies suggesting an increased mTOR activation in this compartment,^45^ as well as the reported importance of mTOR signaling for its regeneration.^46^ In line with the spatial proteomic results, several established pericentral markers such as GS or cytochrome P450 2E1 were decreased in PiZ-Raptor^Δhep^ mice. Given the importance of GS for glutamine production and ammonia detoxification, we quantified blood ammonia levels in PiZ-Raptor^Δhep^ animals and found that they are greatly increased. This finding provides a plausible explanation for the lethal phenotype, despite the presence of only mild liver fibrosis and a largely preserved production of secreted hepatocellular proteins. In fact, the hyperammonemia observed in our animals is reminiscent of inborn errors of metabolism, such as urea cycle disorders, that can rapidly lead to death due to ammonia neurotoxicity.^47^ In fact, in addition to the decrease in GS, we observed alterations in several urea cycle–associated proteins in PiZ Raptor^Δhep^ livers. Further studies are needed to delineate the contribution of the different metabolic pathways to the observed hyperammonemia.

The observations made during our studies show that hepatocellular ablation of mTOR signaling constitutes a double-edged sword, and its down-stream consequences may differ from actions elicited by pharmaceutical inhibitors that yielded positive results in PiZ mice.^12^^,^^15^ This is in line with previous studies demonstrating that mTORC1 ablation as well as pharmaceutical MTOR inhibition via rapamycin transiently reduced hepatosteatosis but increased inflammation and tumorigenesis in the long term.^48^ Similarly, mTOR ablation had a transient beneficial effect in liver-specific autophagy-deficient Atg5 knockouts but had negative consequences in the long term.^49^ Finally, mice with hepatocyte-specific mTOR ablation displayed steatosis and were more susceptible to ischemia/reperfusion injury.^50^ Collectively, these findings suggest that mTOR inhibition should be used with caution, especially in context of pre-existing liver disease. The same applies to novel mTOR inhibitors that are being actively developed for treatment of various tumors.^51^

## Materials and Methods

### Animal Studies

Transgenic mice that overexpress the human Z variant of *SERPINA1* gene (PiZ mice)^52^ were crossbred with mTOR-floxed,^53^ RAPTOR-floxed, or RICTOR-floxed animals (#013188 and #020649; The Jackson Laboratory). To generate a hepatocyte-specific KO mouse, all 3 lines were mated with mice expressing albumin Cre recombinase.^54^ To assess the importance of CHOP in the observed liver injury, hepatocyte-specific RAPTOR KOs were crossbred with constitutive CHOP KO (CHOP-KO) mice^55^ (#005530; The Jackson Laboratory). All animals were maintained on 12-hour dark–light cycles and kept on a standard diet (Ssniff) in the animal facility of RWTH Aachen University. All breeding was performed according to the German guidelines for animal research and animal welfare and was approved by the responsible institution of the state of North Rhine-Westphalia (LANUV). Animal studies were carried out according to Animal Research: Reporting of In Vivo Experiments (ARRIVE) guidelines. Sex-matched animals were sacrificed at the age of 8 weeks, and liver tissue was cut into pieces and transferred to 4% formalin for paraffin embedding and histologic examinations or stored in RNAlater solution (Thermo Fisher Scientific) for RNA isolation. For cutting of frozen sections, tissue was embedded in TissueTek compound in a Tissue Tek Cryomold (Sakura). For protein isolation or caspase-3 assay, tissue was immediately submerged in liquid nitrogen with subsequent storage at −80 °C. Serum was analyzed at the laboratory diagnostic center of the University Hospital Aachen. Blood ammonia levels were measured with the DRI-CHEM NX10N blood ammonia analyzer, using the FUJI DRI-CHEM SLIDE NH3 -WII (Fujifilm) according to the manufacturer’s instructions.

### Quantitative Real-Time Polymerase Chain Reaction Analysis

Tissue samples were stored in RNAlater solution (Thermo Fisher Scientific), and RNA was isolated with the RNAeasy mini kit (Qiagen; ref. 74,104) following the manufacturer’s instructions. For the transcription of RNA to complementary DNA, the M-MLV-Reverse-Transcriptase Kit (Promega) was used. RT-qPCR was performed using the e 300 fast real-time PCR system (Applied Biosystems) with the SYBR Green qRT-PCR mastermix (Thermo Fisher Scientific) and specific primers (Supplementary Table 3). All PCR reactions were performed in duplicate, and the mean expression (Ct values) was used to calculate the relative expression of target gene compared with mouse ribosomal protein L7 (L7), which was used as an internal control.

### Protein Analysis

For the isolation of total proteins, frozen liver tissues were homogenized with the TissueLyser II (Qiagen) in homogenization buffer containing 3% sodium dodecyl sulfate (SDS; Carl Roth) supplemented with protease-inhibitor cOmplete (Roche) and phosphatase-inhibitors PhosStop (Roche). Protein extracts were diluted in 4× reducing Laemmli buffer, and the proteins were separated by 8% to 12% SDS–polyacrylamide gel electrophoresis. Gels were either used for Coomassie staining (Serva) or immunoblotting. For blotting, the proteins were transferred to a methanol-activated polyvinylidene fluoride membrane and incubated with the appropriate primary and secondary antibodies, the latter being conjugated with horseradish peroxidase (Supplementary Table 4). The labeled proteins were visualized by chemiluminescent detection, using enhanced chemiluminescence luminol reagent or enhanced chemiluminescence luminol prime reagent (GE Healthcare) and the ImageQuant LAS4000 (GE Healthcare).

### Tissue Staining

For hematoxylin and eosin staining, PAS-D staining, and immunohistochemical and immunofluorescence staining, paraffin-embedded tissues were used. Tissues were dehydrated in the Interdisziplinäres Zentrum für Klinische Forschung (IZKF) immunhistochemistry facility at the University Hospital Aachen, embedded in paraffin and cut into 2-μm thick sections with the Rotary microtome Epredia HM 325 (Thermo Fisher Scientific). Before staining, the sections were rehydrated in descending alcohol series. For hematoxylin and eosin staining, the slides were incubated in hematoxylin (Carl Roth) for 5 to 10 minutes and subsequently stained in 0.5% eosin (Merck) for 1.5 to 3 minutes. The specimens were covered with Entellan mounting medium (Merck). For PAS-D staining, the slides were incubated with diastase, followed by a 15-minute incubation in Schiff’s reagent. The nuclei were counterstained with hematoxylin. For immunohistochemical staining, antigen retrieval was performed with sodium citrate retrieval solution (Vector Laboratories). Afterwards, samples were incubated with the primary antibodies at 4 °C overnight (Supplementary Table 4) and after washing with biotinylated secondary antibody for 30 minutes at room temperature (RT). Detection was carried out with VECTASTAIN Elite ABC-HRP solution (Vector Laboratories) and the 3,3′-diaminobenzidine substrate kit from Vector Laboratories, without supplementation of nickel. For immunofluorescence staining, primary antibodies (Supplementary Table 4) were added to the slides for 1 hour at RT. After vigorous washing, an incubation with the fluorescently labeled secondary antibody took place for 30 minutes (Supplementary Table 4). For a double staining with 2 primary antibodies from the same host, the first secondary antibody (labeled with Alexa Fluor 488) was diluted 1:100. After washing, slides were blocked with blocking buffer (2% goat serum and 2.5% bovine serum albumin [BSA] in phosphate-buffered saline [PBS]), and the second primary antibody was added for 1 hour at RT (Supplementary Table 4). After washing, the sections were incubated with the second, Alexa Fluor 568–labeled secondary antibody for 30 minutes (Supplementary Table 4). Slides were mounted with ProLong Gold antifade containing 4′,6-diamidin-2-phenylindol (Thermo Fisher Scientific). For TUNEL, tissue was embedded in TissueTek compound (Sakura), snap-frozen in liquid nitrogen, and cut into 5-μm sections. The In Situ Cell Death Detection Kit, Fluorescein from Roche was used, according to the manufacturer’s instructions. Slides were mounted with ProLong Gold antifade containing 4′,6-diamidin-2-phenylindol for the visualization of the nuclei (Thermo Fisher Scientific). Image visualization was carried out with the microscopes Axio Imager A2 or Axio Imager Z1 (Carl Zeiss), and images were analyzed with AxioVision Rel 4.8 software (Carl Zeiss). For analysis of nucleus and cell size, the open-source software QuPath was used.^56^ Cell counting was done with ImageJ.^57^

### Biochemical Assays

To quantify the amount of active caspase-3 in liver tissue, caspase-3 fluorogenic substrate Ac-DEVD-AFC (Enzo Biochem) was used as recommended by the supplier. Each sample was measured in duplicate at the enzyme-linked immunosorbent assay reader μQuant (BioTek Instruments). Excitation was assessed at 390 nm and emission at 510 nm.

### Liver Proteomics

Five individual livers per genotype were lysed, digested, and fractionated as described previously.^58^ After lyophilization, the individual fractions were resuspended in 3% formic acid (FA)/5% acetonitrile (ACN) and loaded onto a nanoLC system (RSLCnano, Thermo Scientific). The peptides were first trapped on a trapping column (Acclaim PepMap100, C18, 5 μm, 100 Å, 300 μm i.d. × 5 mm; Thermo Scientific) and then separated on an analytical column at 45 °C (Easyspray 50 cm column; ES803; Thermo Scientific). Peptide separation was carried out using a 165-minute gradient 0 to 10 minutes: 5% buffer B (buffer A: 0.1% FA; buffer B: 80% ACN, 0.1% FA), 10 to 100 minutes: 5% to 25% buffer B, 100 to 138 minutes: 25% to 35% buffer B, 138 to 141 minutes: 35% to 99% buffer B, 141 to 146 minutes: 99% buffer B, 146 to 149 minutes: 99.5% buffer B, 149 to 165 minutes: 5% buffer B. The mass spectrometry analyses were performed on a Q Exactive plus mass spectrometer (Thermo Scientific) in data-dependent mode. Spray settings were: spray voltage: 2 kV; capillary temperature: 250 °C. MS settings were: 70,000 resolution; AGC target, 1 × 10^6^; maximum injection time, 50 milliseconds; scan range, 350 to 1600 m/z. dd-MS2 settings were: 17,500 resolution; AGC target: 1 × 10^5^; maximum injection time: 55 milliseconds; top 20 precursor fragmentation; isolation window, 2.0 m/z; collision energy, 27. dd settings were: minimum AGC, 5 × 10^2^; 20-second dynamic exclusion; only 2+ to 5+ peptides.

Analysis of all raw mass spectrometry data was carried out using MaxQuant (2.2.0.0) with the built-in Andromeda search engine.^59^ The search was conducted against the *Mus musculus* UniProt database version 08/2023 (only reviewed and canonical sequences) with the manually added mutated AAT Z-variant (accession: NM_001127701.1(SERPINA1):c.1096G>A (p.Glu366Lys)) using MaxQuant default settings. The specific settings were: protease: trypsin (2 missed cleavages); fixed modification: carbamidomethylation; variable modifications: oxidation (Met) and N-terminal protein acetylation. FDR: 0.01 on both protein and peptide spectrum match levels; minimum peptide length: 7 amino acids. Quantification was done using the label-free quantitation algorithm from MaxQuant. Precursor intensities were normalized and were assembled to proteins using DirectLFQ.^60^

### Serum Proteomics

For serum proteome analysis, samples were prepared as described.^32^ Briefly, 5 sera per condition were separated by SDS-polyacrylamide gel electrophoresis using gradient gels (4%–20%; Serva) and stained with Coomassie Brilliant Blue. Five gel slices per lane were excised and proteolytically digested as described.^61^ The lyophilized peptide samples from the individual gel slices were resuspended in 15 μL 3% FA/5% ACN and then loaded onto an liquid chromatography system (RSLCnano, Thermo Scientific) and trapped as above. Sample separation was carried out using an analytical column at 45 °C (Easyspray 25 cm column; ES802; Thermo Scientific) and eluted on to a Q Exactive plus mass spectrometer (Thermo Scientific) with the following 55-minute gradient: gradient 0 to 10 minutes: 5% buffer B (buffer A: 0.1% FA; buffer B: 80% ACN, 0.1% FA), 10 to 30 minutes: 5% to 35% buffer B, 30 to 33 minutes: 35% to 99% buffer B, 33 to 38 minutes: 99% buffer B, 38 to 41 minutes: 99.5% buffer B, 41 to 55 minutes: 5% buffer B. The settings of the mass spectrometer were as above except for the use of top 10 precursor fragmentation. Raw data analysis was conducted as described above.

### Preprocessing of Bulk and Serum Proteomic Datasets

Before any processing, 7412 proteins were identified in the bulk data, and 550 proteins were identified in the serum proteomic data. All entries mapped to the reverse decoy database, and features identified only via peptides comprising a post-translational modification site, were removed. The proteomic data were then log2-transformed and filtered for parameters reaching at least 50% valid values within at least 1 defined subgroup (*Cre+, PiZ Cre+, Raptor*^*Δhep*^*, PiZ Raptor*^*Δhep*^) (bulk data: 6797 proteins; serum data: 327 proteins). The remaining missing values were imputed column-wise with random draws from a distribution of the leftmost tail of the data (based on a quantile regression) [QRILC algorithm].^62^ Subsequently, the values were median-centered.

### Downstream Analysis of Proteomic Data

Differential abundance analysis for proteins was performed by fitting the intensities in linear models with empirical Bayes moderation without the addition of covariates. *P* values were 2-tailed and FDR-adjusted. Features were considered significantly differentially abundant across a given condition if the FDR was <0.05. Results are presented as volcano plots, plotting log2-fold changes on the x-axis and the obtained −log10 *P* values on the y-axis. The signatures of altered features in mouse sera were mapped to publicly available human single-cell RNA sequencing and cellular indexing of transcriptomes and epitopes sequencing data on 10 healthy murine livers^63^ that were accessed via www.livercellatlas.org.

### Targeted Metabolomics of Liver Samples

One hundred milligrams of wet-weight liver tissue was placed into a 5-fold volume of metabolite extracting solution (ACN/water [1/1]) and homogenized with 3 to 5 steel balls for 10 minutes at 30 Hz in a tissue slicer. After 2 minutes of centrifugation at 14,000 rpm, the supernatant-containing metabolites were recovered and stored at −80 °C.

The targeted quantification of amino acids/biogenic amines was performed with the MxP Quant p180 kit (Biocrates Life Science) following the manufacturer’s instructions in a 96-well plate format. Seven calibration standards and 3 different QC standards (QC1, QC2, QC3) were part of the Kit. An additional 4 QC2 samples were distributed over the plate. Ten microliters of extracted samples were added to an adsorption pad system and dried 30 minutes under nitrogen flow. They were derivatized with 50 μL 5% phenylisothiocyanate for 20 minutes and dried for 60 minutes under nitrogen flow. Three hundred microliters of extraction solvent (5 mM ammonium acetate in methanol) was added, and the mixture was shaken for 30 minutes. The samples were then eluted into the lower well by centrifugation for 2 minutes at 100 × g. The extracted elutes were separated into 2 parts, and 1 of them was diluted for liquid chromatography measurement. The amino acids and biogenic amines were assessed by a Hybrid system of a QTRAP mass spectrometer applying electrospray ionization (positive mode) (ABI Sciex API5500Q-TRAP). The mass spectrometer was coupled to an ultra-performance liquid chromatography (Waters Acquity, Waters Corporation), and the liquid chromatography separation was carried out by a hyphenated reverse phase column (Agilent, Zorbax Eclipse XDB C18, 3.0 9100 mm, 3.5 μm; Agilent), preceded with a precolumn (Security Guard, Phenomenex, C18, 4 9 3 mm; Phenomenex) via applying a gradient of solvent A (FA 0.2% in water) and solvent B (FA 0.2% in acetonitrile) over 7.3 minutes (0.5 minutes 0% B, 5 minutes 70% B, 0.3 minutes 70% B, 2 minutes 0% B) at a flow rate of 500 μL/min and a temperature of 50 °C. Raw data of the Analyst software (AB Sciex) were processed by the MetIDQ software, which is an integrated part of the p180 Kit (Biocrates Life Sciences AG). For fully quantitative measurements of the p180 Kit, the lower limit of quantification was determined in plasma experimentally by Biocrates Life Sciences AG.

### Spatial Proteomics

#### Immunofluorescence staining and imaging

Deep Visual Proteomics experiments were conducted as previously reported.^64^ Two micrometer polyethylene naphthalate membrane slides were pretreated with ultraviolet ionization for 1 hour at 254 nm. Immediately thereafter, the slides were washed consecutively for 5 minutes each in a mixture of 7 mL VECTABOND reagent filled up to 350 mL with acetone, followed by a 30-second wash in ultrapure water before being dried with a gentle nitrogen airflow. For sectioning, tissue blocks were placed in a cryostat (Leica CM3050) set to −18 °C for the chamber and −15 °C for the object temperature, allowing them to equilibrate for 30 minutes. The blocks were then trimmed, and sections were cut to a thickness of 10 μm using a disposable high-profile blade (Leica 818). The frozen sections were transferred to pretreated, cold polyethylene naphthalate membrane slides and melted for less than 5 seconds on an RT surface. Sections were fixed in prewarmed 4% paraformaldehyde in PBS at 37 °C, then in 95% ethanol at RT, and again in 4% paraformaldehyde in PBS at 37 °C. Slides were rinsed in PBS and incubated in a 5% BSA–PBS blocking solution for 1 hour before staining. Staining was performed for 1 hour at 37 °C in a humid, dark chamber using 200 μL of a 1-step liver painting in 1% BSA, including 1:1000 Hoechst, and/or the respective antibodies (Supplementary Table 4). Slides were washed 3 times for 2 minutes in PBS in the dark, mounted with 21 FL ProLong Diamond mounting medium (Invitrogen; P36961), and covered with a 22 × 22 mm #1.5 coverslip. Slides were stored in 50-mL tubes with desiccating material at 4 °C until imaging. Full-section images were recorded on an Axioscan 7 (Zeiss) with a plan-apochromat 20×/0.8 M27 objective and the Axiocam 712 in 4 fluorescence channels: 385 nm, 475 nm, 567 nm, and 630 nm excitation, each at 50% light source intensity. Exposure times were optimized to keep the signal within the dynamic acquisition range. Pixels were binned in 2 for a final scaling of 0.345 μm × 0.345 μm.

#### Laser microdissection

Images were imported into the Biological Image Analysis Software (Single-Cell Technologies Ltd)^33^ using the provided import tool. Hepatocytes were identified through a deep neural network designed for histologic cytoplasm segmentation, based on phalloidin staining with a spatial scaling input of 1.2, a detection confidence of 40%, and a contour confidence of 30%. Contours within the range of 135 μm^2^ to 1350 μm^2^ were considered, without any additional exclusion criteria. After supervised machine learning removed duplicates and false identifications, contours were exported without any shape offset, along with 3 calibration points selected at characteristic tissue positions. For 5-shape proteomes, adjacent shapes forming a pentagon-like structure were manually selected. Single shapes were picked randomly, and every 15th to 25th shape was assigned to adjacent wells in a 384-well plate. Contour outlines were imported following reference point alignment, and 100 shapes per sample were cut using laser microdissection with the LMD7 (Leica) in a semiautomated manner. The settings were: power 59, aperture 1, speed 60, middle pulse count 1, final pulse −1, head current 48% to 52%, pulse frequency 3282, and offset 100. The shapes were sorted into a low-binding 384-well plate (Eppendorf; 0,030,129,547) with an empty well between samples. Plates were sealed, centrifuged at 1000 × g for 5 minutes, and then frozen at −20 °C until further processing.

#### Peptide preparation

Peptides were prepared semiautomatically using a Bravo pipetting robot (Agilent). To prevent evaporation during each incubation step, the plates were tightly sealed with 2 stacked aluminum lids (Thermo Fisher Scientific; AB0626). Initially, the plates were removed from the freezer and centrifuged. The wells were washed on the robot with 28 μL of 100% ACN and dried in a SpeedVac (Eppendorf) at 45 °C for 20 minutes. The shapes were then resuspended in 6 μL of 60 mM triethylammonium bicarbonate buffer (pH 8.5, Sigma) with 0.013% n-dodecyl-β-D-maltoside (Sigma) and heated for 30 minutes at 95 °C in a PCR cycler with a lid temperature of 110 °C. After adding 1 μL of 80% ACN (to achieve a final concentration of 10%), the samples were incubated for another 30 minutes at 75 °C, briefly cooled, and then 1 μL containing 4 ng LysC and 6 ng trypsin was added. The samples were digested for 18 hours and then acidified to 1% trifluoroacetic acid.

#### Liquid chromatography–tandem mass spectrometry analysis of single shapes

Samples were analyzed using the Evosep One liquid chromatography system (EvoSep) connected to a timsTOF SCP mass spectrometer (Bruker Daltonics). The Whisper40 samples per day method was employed with the Aurora Elite CSI third generation 15-cm, 75-μm ID (AUR3-15075C18-CS IonOpticks) at 50 °C within a nanoelectrospray ion source (Bruker Daltonics). The mobile phases used were 0.1% FA in liquid chromatography–mass spectrometry-grade water (buffer A) and 99.9% ACN/0.1% FA (buffer B). The timsTOF SCP was operated using an optimal dia–parallel accumulation-serial fragmentation method, generated with Python tool py_diAID,^65^ which included 8 dia-parallel accumulation-serial fragmentation scans with variable width and 2 ion mobility windows per scan, covering an m/z range from 300 to 1200 and an ion mobility range from 0.7 to 1.3 V s cm^−2^. The mass spectrometer was set to high-sensitivity mode, with an accumulation and ramp time of 100 ms, a capillary voltage of 1400 V, and a collision energy that ramped linearly from 20 eV at 1/K_0_ = 0.6 V s cm^−2^ to 59 eV at 1/K_0_ = 1.6 V s cm^−2^.

#### Spectral search and data analysis

All files were analyzed collectively using DIA-NN (version 1.8.1)^66^ against a predicted library. The parameters included a mass and MS1 mass accuracy of 15.0, scan windows of 9, and activated isotopologues, with match-between-runs and heuristic protein inference enabled and no shared spectra, in single-pass mode. Proteins were inferred from genes. Library generation was configured to ‘IDs, RT & IM profiling,’ and ‘Robust LC (high precision)’ was used as the quantification strategy. The precursor intensities were normalized and assembled to proteins using directLFQ at standard settings.^60^ Proteomics data analysis was conducted using R v4.2.2, and statistical testing was performed with the limma package. All reported *P* values were corrected for multiple testing using the “fdr” adjustment method.

### Statistical Analysis

Statistical analysis was done with the software Graph Pad Prism 8 (GraphPad Software). The Shapiro–Wilk test was performed to check the distribution of the results. For normally distributed data, an unpaired 2-tailed *t* test with Welch’s correction was done. Samples that were not normally distributed were tested with a nonparametric 2-tailed Mann–Whitney test. For multigroup comparisons, a parametric 1-way analysis of variance with Sidak’s test or nonparametric analysis of variance with Kruskal–Wallis post hoc test were used. For the comparison of survival curves, a log-rank (Mantel–Cox) test was carried out. Two-sided *P* values were labeled with asterisks to indicate ∗*P* ≤ .05, ∗∗*P* ≤ .01, ∗∗∗*P* ≤ .001, and ∗∗∗∗*P* ≤ .0001. Statistical analysis of Proteomics data were conducted using the R environment^67^ (R Foundation; version 4.4.1) and R Studio^68^ (version 2024.04.2), employing the following software packages: *dplyr,*^69^ *ggplot2,*^70^ *imputeLCMD,*^71^ *preprocessCore,*^72^ *limma,*^73^ *SummarizedExperiment,*^74^ *circlize,*^75^ *and ComplexHeatmap.*^76^ PCA was carried out for the preprocessed proteomic data using the base R *prcomp* function with default parameters. To aid the visualization, 95% confidence interval ellipses were generated assuming a multivariate *t* distribution.

## Supplementary Material

Supplementary Tables 1–4

Extended PDF
