# Supplementary material for: Loss of the Mechanistic Target of Rapamycin Complex 1 Causes a Lethal Alpha-1 Antitrypsin Deficiency–Associated Liver Disease
Source: Cell Mol Gastroenterol Hepatol. 2026 Jun 4;20(10):101824. doi: 10.1016/j.jcmgh.2026.101824 (PMC13543902; doi:10.1016/j.jcmgh.2026.101824)
Supplement: Supplementary file 1 [file mmc1.pdf]

Supplementary Table 1. Increased and decreased serum proteins in PiZ RΔhep mice compared to all other groups.

uniprot	gene symbol	log2FC	p-value	FDR	hepatocellular yes/no	subcellular location
P42703	Lifr	-3.6025	9.16E-08	2.99E-05	YES	intracellular
Q01279	Egfr	-2.9952	6.60E-07	1.08E-04	YES	intracellular
Q5FW60	Mup20	-8.2378	1.58E-06	1.72E-04	YES	secreted
P28665	Mug1	-2.3658	3.37E-06	2.75E-04	YES	secreted
A2BIM8	Mup9	-5.1774	4.72E-06	3.09E-04	NO	
P52430	Pon1	-2.0940	5.73E-06	3.12E-04	YES	secreted
P34928	Apoc1	-1.9754	7.01E-06	3.27E-04	YES	secreted
O55234	Psmb5	2.2992	2.31E-05	9.46E-04	YES	intracellular
P02772	Afp	6.6116	2.66E-05	9.68E-04	NO	
Q61838	A2m	-1.3823	4.52E-05	1.14E-03	NO	
Q8CG14	C1sa	-1.6637	3.84E-05	1.14E-03	NO	
P06909	Cfh	-1.3602	4.45E-05	1.14E-03	YES	secreted
Q60692	Psmb6	1.7363	3.90E-05	1.14E-03	YES	intracellular
P06684	C5	-1.3600	5.98E-05	1.15E-03	NO	
Q9R1P3	Psmb2	1.1911	5.92E-05	1.15E-03	YES	intracellular
P31532	Saa4	-3.8129	5.40E-05	1.15E-03	YES	secreted
Q8CGR7	Upp2	-2.3838	5.69E-05	1.15E-03	YES	intracellular
P26262	Klkb1	-1.3074	7.53E-05	1.32E-03	YES	secreted
P07759	Serpina3k	-1.5472	7.67E-05	1.32E-03	YES	secreted
P01029	C4b	-1.3731	9.77E-05	1.60E-03	YES	secreted
Q9CPY7	Lap3	5.3593	1.08E-04	1.69E-03	YES	intracellular
Q8BH35	C8b	-1.8163	1.17E-04	1.75E-03	YES	intracellular
Q9QWK4	Cd5l	1.4284	1.39E-04	1.97E-03	YES	intracellular
Q8CFG8	C1sa	-2.2657	1.51E-04	2.00E-03	NO	
Q8K182	C8a	-2.1166	1.53E-04	2.00E-03	YES	intracellular
Q60590	Orm2	-1.5728	1.83E-04	2.30E-03	NO	
Q91Y97	Aldob	2.3843	2.25E-04	2.53E-03	YES	intracellular
E9Q414	Apob	-1.2575	2.10E-04	2.53E-03	YES	intracellular
P33622	Apoc3	-1.9097	2.24E-04	2.53E-03	YES	secreted
P99026	Psmb4	1.1583	2.48E-04	2.70E-03	YES	intracellular
Q9R1P4	Psma1	1.1618	2.94E-04	3.10E-03	YES	intracellular
O70362	Gpld1	-1.9103	3.20E-04	3.27E-03	YES	secreted
Q05020	Apoc2	-1.3887	4.11E-04	3.77E-03	NO	
P29391	Ftl1	3.7475	3.99E-04	3.77E-03	YES	intracellular
A6X935	Itih4	-1.8165	3.87E-04	3.77E-03	YES	secreted
P20918	Plg	-1.8196	4.15E-04	3.77E-03	YES	secreted
Q9ESB3	Hrg	-1.1315	4.79E-04	4.23E-03	YES	secreted

P97290	Serping1	-1.7834	4.98E-04	4.28E-03	YES	secreted
Q9DBB9	Cpn2	-1.2590	5.24E-04	4.29E-03	YES	secreted
O70435	Psma3	1.2524	5.16E-04	4.29E-03	YES	intracellular
P49722	Psma2	3.5365	6.27E-04	5.00E-03	YES	intracellular
P01725		1.9251	6.77E-04	5.27E-03	NO	
P33587	Proc	-0.9532	7.51E-04	5.71E-03	YES	intracellular
P70195	Psmb7	2.6594	7.73E-04	5.74E-03	YES	intracellular
P70389	Igfals	-2.2536	9.20E-04	6.68E-03	YES	secreted
Q61702	Itih1	-0.8420	1.17E-03	8.35E-03	YES	secreted
P62984	Uba52	-0.9358	1.42E-03	9.88E-03	YES	intracellular
Q9R1P0	Psma4	1.1458	1.79E-03	1.22E-02	YES	intracellular
P47878	Igfbp3	-3.5255	1.84E-03	1.23E-02	YES	secreted
Q9JHH6	Cpb2	-1.0199	1.88E-03	1.23E-02	YES	secreted
P09813	Apoa2	-1.4429	2.25E-03	1.31E-02	YES	secreted
Q8CG16	C1ra	-1.1767	2.23E-03	1.31E-02	YES	secreted
Q9JJN5	Cpn1	-0.9009	2.18E-03	1.31E-02	YES	secreted
P97807	Fh	2.5362	2.09E-03	1.31E-02	NO	
P01942	Hba	-1.0490	2.10E-03	1.31E-02	NO	
P26928	Mst1	-3.6644	2.20E-03	1.31E-02	YES	secreted
P04186	Cfb	-1.0720	2.92E-03	1.68E-02	YES	secreted
P07361	Orm2	-1.7256	3.16E-03	1.78E-02	NO	
P06683	C9	-1.8142	3.48E-03	1.89E-02	YES	intracellular
P35441	Thbs1	-1.2469	3.42E-03	1.89E-02	NO	
P84750		1.4204	3.93E-03	2.07E-02	NO	
O09164	Sod3	0.8391	3.89E-03	2.07E-02	YES	intracellular
P01837		1.0488	4.22E-03	2.19E-02	NO	
P01630		1.2007	4.39E-03	2.19E-02	NO	
P01027	C3	-0.8478	4.41E-03	2.19E-02	YES	secreted
Q8CIZ8	Vwf	-1.0582	4.42E-03	2.19E-02	NO	
P04939	Mup20	-4.2629	4.54E-03	2.21E-02	YES	secreted
P01674		1.7042	4.85E-03	2.33E-02	NO	
P01898	H2-Q10	-1.2712	4.94E-03	2.34E-02	YES	intracellular
P35505	Fah	1.2851	5.12E-03	2.39E-02	YES	intracellular
P21180	C2	-1.3159	5.72E-03	2.60E-02	YES	secreted
Q80YC5	F12	-1.0095	5.89E-03	2.60E-02	YES	secreted
O09159	Man2b1	-0.8506	5.85E-03	2.60E-02	YES	intracellular
P98064	Masp1	-0.9919	5.74E-03	2.60E-02	YES	secreted
P97449	Anpep	1.0643	6.15E-03	2.68E-02	NO	
Q60994	Adipoq	0.8126	6.33E-03	2.69E-02	NO	
Q08879	Fbln1	-1.4445	6.25E-03	2.69E-02	NO	
Q61730	Il1rap	-1.1802	6.48E-03	2.71E-02	YES	intracellular
P16294	F9	-2.6061	6.85E-03	2.80E-02	YES	secreted

P07758	Serpina1a	-0.9984	6.81E-03	2.80E-02	YES	secreted
P13707	Gpd1	2.8804	7.00E-03	2.83E-02	YES	intracellular
P01887	B2m	-0.8453	7.45E-03	2.95E-02	YES	secreted
Q9Z2U0	Psma7	0.9309	7.48E-03	2.95E-02	YES	intracellular
P11276	Fn1	-0.8827	7.78E-03	3.03E-02	YES	secreted
P01750	Ighv1-62-3	4.2969	8.47E-03	3.26E-02	NO	
P29788	Vtn	-0.7168	8.67E-03	3.30E-02	YES	secreted
O09061	Psmb1	1.1878	9.09E-03	3.42E-02	YES	intracellular
Q9R1P1	Psmb3	3.2264	9.50E-03	3.53E-02	YES	intracellular
Q61176	Arg1	3.4599	9.64E-03	3.54E-02	YES	intracellular
P62897	Cycs	1.7797	9.91E-03	3.60E-02	YES	intracellular
Q64442	Sord	1.2338	1.03E-02	3.72E-02	YES	intracellular
P19221	F2	-0.6777	1.07E-02	3.79E-02	YES	secreted
P41317	Mbl2	-0.6805	1.15E-02	4.01E-02	YES	secreted
P97298	Serpinf1	-0.8972	1.15E-02	4.01E-02	YES	secreted
Q8BH61	F13a1	-3.1901	1.21E-02	4.16E-02	NO	
P28076	Psmb9	2.1533	1.36E-02	4.62E-02	YES	intracellular
P18531	Ighv3-6	3.7638	1.38E-02	4.64E-02	NO	
Q07235	Serpine2	-1.6752	1.40E-02	4.68E-02	NO	
P11680	Cfp	1.0898	1.50E-02	4.97E-02	YES	secreted
P01645		0.7565	1.90E-02	6.20E-02	NO	
Q61646	Hp	-1.8378	1.92E-02	6.20E-02	YES	secreted
P0DP28	Calml3	-0.7423	1.98E-02	6.31E-02	NO	
O88844	Idh1	3.7041	1.99E-02	6.31E-02	YES	intracellular
P01806		3.0024	2.06E-02	6.34E-02	NO	
Q9Z1R3	Apom	-1.3321	2.05E-02	6.34E-02	YES	secreted
Q61805	Lbp	-1.5891	2.05E-02	6.34E-02	YES	intracellular
Q00898	Serpina1b	-1.6623	2.07E-02	6.34E-02	YES	secreted
P28063	Psmb8	1.3850	2.24E-02	6.78E-02	YES	intracellular
Q8CIF4	Btd	1.7244	2.35E-02	7.01E-02	NO	
Q91X72	Hpx	-1.0142	2.36E-02	7.01E-02	YES	secreted
Q8R555	Crtac1	-1.7992	2.51E-02	7.40E-02	NO	
P06330		1.1331	2.65E-02	7.66E-02	NO	
P09470	Ace	-0.6316	2.63E-02	7.66E-02	NO	
Q8VCG4	C8g	-1.3473	2.68E-02	7.68E-02	YES	intracellular
Q9Z126	Pf4	-0.9294	2.73E-02	7.69E-02	YES	secreted
P60867	Rps20	1.4407	2.71E-02	7.69E-02	YES	intracellular
P13609	Srgn	-1.4500	2.75E-02	7.69E-02	YES	intracellular
P03977		2.9134	3.03E-02	8.35E-02	NO	
Q8R4G0	Ntng1	-4.1087	3.04E-02	8.35E-02	NO	
P70274	Sepp1	0.6858	3.12E-02	8.52E-02	NO	
Q00724	Rbp4	-1.0477	3.22E-02	8.69E-02	YES	secreted

P08228	Sod1	-1.5571	3.34E-02	8.94E-02	YES	intracellular
Q9QXF8	Gnmt	3.5431	3.43E-02	9.06E-02	YES	intracellular
Q9R098	Hgfac	-1.1411	3.42E-02	9.06E-02	YES	secreted
Q64726	Azgp1	-0.6069	3.55E-02	9.13E-02	YES	secreted
P05201	Got1	2.2035	3.54E-02	9.13E-02	YES	intracellular
P01872	Ighm	0.7413	3.50E-02	9.13E-02	YES	intracellular
Q3UVD5	Lgr6	-3.1570	3.67E-02	9.36E-02	NO	
P18760	Cfl1	-1.9481	3.75E-02	9.37E-02	YES	intracellular
P10649	Gstm1	2.8934	3.75E-02	9.37E-02	YES	intracellular
P49429	Hpd	2.7646	3.74E-02	9.37E-02	YES	intracellular
P03953	Cfd	0.6287	3.90E-02	9.65E-02	NO	
Q8VCR7	Abhd14b	-2.6264	3.96E-02	9.73E-02	YES	intracellular
P01749	Ighv1-62-3	1.6332	4.09E-02	9.98E-02	NO	
Q61268	Apoc4	-1.2108	4.36E-02	1.06E-01	YES	secreted
P01747		3.2921	4.49E-02	1.07E-01	NO	
Q06890	Clu	-0.4906	4.49E-02	1.07E-01	YES	intracellular
Q91ZX7	Lrp1	-1.4106	4.54E-02	1.08E-01	YES	intracellular
P13020	Gsn	-0.8884	4.60E-02	1.08E-01	YES	intracellular
P01639	Gm5571	1.0953	4.65E-02	1.09E-01	NO	
Q61147	Cp	-0.7922	4.78E-02	1.11E-01	YES	secreted
Q61704	Itih3	-0.6352	4.91E-02	1.13E-01	YES	secreted
P07309	Ttr	-0.6147	5.04E-02	1.15E-01	YES	secreted
P01843		1.0496	5.09E-02	1.16E-01	NO	
Q00623	Apoa1	-0.5501	5.17E-02	1.17E-01	YES	secreted
Q9Z2U1	Psma5	1.2029	5.23E-02	1.17E-01	YES	intracellular
P17182	Eno1	2.4853	5.29E-02	1.18E-01	YES	intracellular
P01638		0.8124	5.43E-02	1.20E-01	NO	
O88947	F10	-0.5347	5.48E-02	1.20E-01	YES	secreted
Q00897	Serpina1d	-0.8511	5.48E-02	1.20E-01	YES	secreted
P01009	SERPINA1	2.4990	5.52E-02	1.20E-01	NO	
P16858	Gapdh	-1.3324	5.57E-02	1.20E-01	YES	intracellular
P08905	Lyz2	-0.4985	5.79E-02	1.24E-01	YES	secreted
P18524		2.3681	6.06E-02	1.28E-01	NO	
O70165	Fcn1	0.5281	6.13E-02	1.28E-01	NO	
P02088	Hbb-b1	-0.6340	6.01E-02	1.28E-01	NO	
P01592	Igj	0.9647	6.09E-02	1.28E-01	NO	
Q9QUM9	Psma6	2.1240	6.16E-02	1.28E-01	YES	intracellular
P82198	Tgfbi	2.5988	6.50E-02	1.34E-01	YES	secreted
P12246	Apcs	0.9410	6.82E-02	1.39E-01	YES	secreted
Q02053	Uba1	-1.5650	7.00E-02	1.42E-01	YES	intracellular
P01631		0.6086	7.16E-02	1.43E-01	NO	
Q9CQW3	Proz	-1.5434	7.19E-02	1.43E-01	YES	secreted

O88968	Tcn2	-0.4459	7.14E-02	1.43E-01	YES	secreted
P01654		0.9082	7.52E-02	1.49E-01	NO	
P01728		0.8440	7.76E-02	1.53E-01	NO	
P00329	Adh1	2.7074	8.56E-02	1.65E-01	YES	intracellular
P01633	Igk-V19-17	0.7693	8.53E-02	1.65E-01	NO	
P49182	Serpind1	-0.6649	8.44E-02	1.65E-01	YES	
P29533	Vcam1	-0.5933	8.47E-02	1.65E-01	YES	intracellular
P03976		2.3845	9.28E-02	1.78E-01	NO	
Q8K0D2	Habp2	2.3768	9.46E-02	1.80E-01	YES	secreted
P04944		1.7498	9.57E-02	1.81E-01	NO	
P08226	Apoe	-0.4681	9.67E-02	1.82E-01	YES	intracellular
O89020	Afm	-0.5916	1.05E-01	1.95E-01	YES	secreted
P62702	Rps4x	1.2097	1.05E-01	1.95E-01	YES	intracellular
Q3UV17	Krt76	0.8030	1.07E-01	1.98E-01	NO	
P70375	F7	-1.2502	1.13E-01	2.08E-01	YES	secreted
P01878		1.2109	1.16E-01	2.11E-01	NO	
P05064	Aldoa	0.5627	1.18E-01	2.13E-01	YES	intracellular
Q8VCS0	Pglyrp2	0.6365	1.17E-01	2.13E-01	YES	intracellular
P51885	Lum	-0.5541	1.19E-01	2.13E-01	YES	secreted
P14152	Mdh1	1.5667	1.22E-01	2.17E-01	YES	intracellular
Q03311	Bche	-0.8527	1.25E-01	2.21E-01	YES	secreted
P01799		0.7877	1.25E-01	2.22E-01	NO	
P16460	Ass1	2.4719	1.28E-01	2.22E-01	YES	intracellular
P01897	H2-L	-0.5769	1.28E-01	2.22E-01	NO	
P39039	Mbl2	-0.4317	1.28E-01	2.22E-01	YES	secreted
P28666	Mug2	-1.1337	1.28E-01	2.22E-01	YES	secreted
P61939	Serpina7	3.9534	1.30E-01	2.23E-01	NO	
P18525		1.0330	1.32E-01	2.26E-01	NO	
P46412	Gpx3	0.5851	1.41E-01	2.41E-01	NO	
P01844	Iglc2	0.6338	1.43E-01	2.41E-01	YES	
Q8C7G5	Apoa5	-1.0001	1.44E-01	2.43E-01	YES	intracellular
Q8CGP2	Hist1h2bp	1.5813	1.46E-01	2.45E-01	NO	
P01867	Igh-3	0.7910	1.49E-01	2.49E-01	NO	
P01864		0.7611	1.54E-01	2.55E-01	NO	
P16301	Lcat	-0.4925	1.64E-01	2.72E-01	YES	secreted
P01642	Gm10881	0.8805	1.66E-01	2.74E-01	NO	
P62827	Ran	-0.7250	1.70E-01	2.77E-01	YES	intracellular
P14131	Rps16	-0.6001	1.70E-01	2.77E-01	YES	intracellular
Q9WUU7	Ctsz	0.6485	1.78E-01	2.87E-01	YES	intracellular
P11352	Gpx1	0.6184	1.87E-01	3.01E-01	YES	intracellular
P98086	C1qa	-0.3876	1.89E-01	3.03E-01	YES	secreted
P01786		0.6109	1.92E-01	3.05E-01	NO	

Q9R1E6	Enpp2	1.7852	1.92E-01	3.05E-01	NO	
P17742	Ppia	1.1739	1.94E-01	3.05E-01	YES	intracellular
P18337	Sell	1.8115	1.93E-01	3.05E-01	YES	intracellular
O70570	Pigr	1.4435	1.96E-01	3.07E-01	YES	intracellular
P84244	H3f3a	1.4867	1.97E-01	3.07E-01	YES	intracellular
P01648		0.5108	2.00E-01	3.10E-01	NO	
P01679		0.6569	2.07E-01	3.15E-01	NO	
Q07456	Ambp	-0.3421	2.07E-01	3.15E-01	YES	intracellular
Q06770	Serpina6	0.6799	2.04E-01	3.15E-01	YES	secreted
Q9Z1T2	Thbs4	-1.5394	2.06E-01	3.15E-01	NO	
Q9R097	Spint1	0.9668	2.11E-01	3.19E-01	NO	
Q9ET66	Pi16	-0.4070	2.14E-01	3.22E-01	YES	secreted
P01660		0.6801	2.15E-01	3.23E-01	NO	
Q8R121	Serpina10	-0.5143	2.17E-01	3.23E-01	YES	secreted
Q91WP0	Masp2	-0.3030	2.20E-01	3.28E-01	YES	secreted
P01632	Igkv7-33	0.4352	2.23E-01	3.30E-01	NO	
Q91WP6	Serpina3n	-0.4641	2.30E-01	3.38E-01	YES	secreted
P01798		0.6661	2.36E-01	3.45E-01	NO	
P05366	Saa1	-1.2603	2.36E-01	3.45E-01	YES	secreted
O08709	Prdx6	1.4042	2.39E-01	3.47E-01	YES	intracellular
P22599	Serpina1b	-0.3892	2.41E-01	3.49E-01	YES	secreted
O88783	F5	-0.3230	2.52E-01	3.59E-01	YES	secreted
O35930	Gp1ba	-0.3202	2.52E-01	3.59E-01	NO	
P29621	Serpina3k	-0.5051	2.50E-01	3.59E-01	YES	secreted
P03987		0.4949	2.54E-01	3.61E-01	NO	
P62806	Hist1h4a	1.0021	2.55E-01	3.61E-01	NO	
P11087	Col1a1	-1.5448	2.62E-01	3.67E-01	NO	
P97321	Fap	1.2330	2.61E-01	3.67E-01	NO	
P55284	Cdh5	-0.4661	2.64E-01	3.69E-01	YES	intracellular
Q8BK48	Ces2e	-1.2336	2.69E-01	3.74E-01	YES	unknown
P01635		0.4606	2.70E-01	3.74E-01	NO	
P21614	Gc	-0.3633	2.77E-01	3.83E-01	YES	secreted
P01636		0.4582	2.80E-01	3.84E-01	NO	
Q03734	Serpina3m	-0.4112	2.82E-01	3.86E-01	YES	secreted
Q64727	Vcl	-0.4721	2.85E-01	3.88E-01	YES	intracellular
C0HKE6	Hist1h2ak	1.1716	2.91E-01	3.95E-01	NO	
O08677	Kng1	0.3611	2.98E-01	4.03E-01	YES	secreted
P50247	Ahcy	0.9277	3.12E-01	4.14E-01	YES	intracellular
P29699	Ahsg	0.4051	3.13E-01	4.14E-01	YES	secreted
P51910	Apod	-0.2868	3.08E-01	4.14E-01	NO	
Q9R111	Gda	-0.8489	3.10E-01	4.14E-01	YES	
Q62351	Tfrc	-0.4235	3.10E-01	4.14E-01	YES	intracellular

P07743	Bpifa2	-1.1103	3.26E-01	4.30E-01	NO	
Q61316	Hspa4	1.0832	3.30E-01	4.34E-01	YES	intracellular
P14847	Crp	-0.5750	3.36E-01	4.40E-01	YES	secreted
Q9QXC1	Fetub	-0.3858	3.48E-01	4.53E-01	YES	secreted
Q9JME2	Chst11	1.3769	3.55E-01	4.57E-01	NO	
Q8BPB5	Efemp1	0.6020	3.54E-01	4.57E-01	YES	secreted
Q01853	Vcp	-0.9506	3.53E-01	4.57E-01	YES	intracellular
P00687	Amy1	1.1247	3.61E-01	4.63E-01	YES	secreted
P08113	Hsp90ab1	1.0036	3.63E-01	4.64E-01	YES	intracellular
P10126	Eef1a1	0.9107	3.80E-01	4.83E-01	YES	intracellular
P01680		0.4897	3.87E-01	4.91E-01	NO	
P43025	Clec3b	0.3884	3.95E-01	4.99E-01	NO	
P01629		0.6308	4.08E-01	5.06E-01	NO	
Q61508	Ecm1	1.2163	4.07E-01	5.06E-01	YES	secreted
Q8BTM8	Flna	0.7423	4.06E-01	5.06E-01	YES	intracellular
P09411	Pgk1	-1.0653	4.06E-01	5.06E-01	YES	intracellular
P20065	Tmsb4x	-1.1969	4.09E-01	5.06E-01	YES	intracellular
Q61129	Cfi	-0.2449	4.10E-01	5.07E-01	YES	secreted
P60710	Actb	-0.2360	4.33E-01	5.33E-01	YES	intracellular
P06728	Apoa4	-0.2051	4.43E-01	5.42E-01	YES	secreted
P52480	Pkm	-0.6677	4.48E-01	5.47E-01	YES	intracellular
P09581	Csf1r	-0.3344	4.51E-01	5.48E-01	YES	intracellular
P11859	Agt	0.3685	4.65E-01	5.62E-01	YES	secreted
P10605	Ctsb	0.6625	4.66E-01	5.62E-01	YES	intracellular
Q05909	Ptprg	0.8207	4.68E-01	5.62E-01	YES	intracellular
P62821	Rab1A	-0.5010	4.77E-01	5.69E-01	NO	
Q61247	Serpinf2	-0.2449	4.75E-01	5.69E-01	YES	secreted
E9PV24	Fga	-0.6131	5.05E-01	6.00E-01	YES	secreted
Q8CFX1	H6pd	0.7752	5.18E-01	6.14E-01	YES	intracellular
P23492	Pnp	1.2219	5.35E-01	6.28E-01	YES	intracellular
Q62009	Postn	-0.5278	5.36E-01	6.28E-01	YES	intracellular
Q80YX1	Tnc	0.4596	5.36E-01	6.28E-01	NO	
P39876	Timp3	-0.5727	5.40E-01	6.30E-01	YES	secreted
Q8VDD5	Myh9	0.4566	5.45E-01	6.34E-01	YES	intracellular
Q6GQT1	A2mp	0.6555	5.59E-01	6.48E-01	NO	
Q8BND5	Qsox1	-0.1660	5.81E-01	6.71E-01	YES	intracellular
Q921I1	Tf	-0.2137	5.86E-01	6.75E-01	NO	
P13595	Ncam1	0.6028	5.89E-01	6.76E-01	NO	
P06151	Ldha	0.4761	5.92E-01	6.77E-01	YES	intracellular
P01790		-0.9430	6.31E-01	7.19E-01	NO	
P14106	C1qb	-0.1284	6.57E-01	7.41E-01	YES	secreted
P23953	Ces1c	0.1624	6.57E-01	7.41E-01	YES	intracellular

P35700	Prdx1	0.4155	6.54E-01	7.41E-01	YES	intracellular
Q923D2	Blvrb	0.5480	6.63E-01	7.45E-01	YES	intracellular
Q9WVJ3	Cpq	0.1585	6.70E-01	7.50E-01	YES	intracellular
Q60963	Pla2g7	0.1734	6.76E-01	7.52E-01	YES	secreted
Q61171	Prdx2	-0.2382	6.80E-01	7.52E-01	YES	intracellular
Q80ZX8	Spag1	0.5884	6.77E-01	7.52E-01	NO	
Q8K1I3	Spp2	-0.2157	6.80E-01	7.52E-01	YES	secreted
Q07968	F13b	0.1438	6.87E-01	7.54E-01	YES	secreted
O70456	Sfn	0.2251	6.87E-01	7.54E-01	NO	
P07901	Hsp90aa1	0.5361	6.99E-01	7.65E-01	YES	intracellular
Q02257	Jup	0.3310	7.03E-01	7.66E-01	YES	intracellular
P63260	Actb	0.2028	7.10E-01	7.71E-01	YES	intracellular
P18528		0.3541	7.19E-01	7.77E-01	NO	
Q01339	Apoh	-0.1250	7.20E-01	7.77E-01	YES	secreted
P01902	H2-Q10	0.1328	7.49E-01	8.05E-01	YES	intracellular
P04945		0.2455	7.58E-01	8.10E-01	NO	
P15327	Bpgm	-0.1285	7.75E-01	8.10E-01	NO	
P13634	Ca1	-0.2181	7.58E-01	8.10E-01	NO	
P19096	Fasn	-0.4158	7.68E-01	8.10E-01	YES	intracellular
Q61703	Itih2	0.0821	7.63E-01	8.10E-01	YES	secreted
Q60605	Myl6	-0.2845	7.65E-01	8.10E-01	YES	
Q99LX0	Park7	-0.3264	7.66E-01	8.10E-01	YES	intracellular
Q08761	Pros1	-0.1001	7.70E-01	8.10E-01	YES	secreted
P32261	Serpinc1	0.0800	7.75E-01	8.10E-01	YES	secreted
G5E8Q8	Adgrf5	0.1264	8.32E-01	8.67E-01	YES	intracellular
O08807	Prdx1	0.2592	8.35E-01	8.67E-01	YES	intracellular
Q8VCC2	Ces1c	-0.2878	8.51E-01	8.81E-01	YES	intracellular
P01868	Ighg1	0.0582	8.63E-01	8.87E-01	NO	
P30412	Ppic	0.1671	8.63E-01	8.87E-01	YES	intracellular
P55065	Pltp	-0.1143	8.85E-01	9.07E-01	YES	intracellular
Q99PT1	Arhgdia	0.0543	9.16E-01	9.36E-01	YES	intracellular
Q02105	C1qc	-0.0325	9.21E-01	9.38E-01	YES	secreted
Q9DBD0	Ica	-0.0294	9.29E-01	9.44E-01	NO	
P08607	C4bpa	-0.0300	9.38E-01	9.50E-01	NO	
P00920	Ca2	-0.0216	9.59E-01	9.68E-01	NO	
P01754	Ighv1-62-3	0.0211	9.75E-01	9.81E-01	NO	
Q924A2	Cic	0.0187	9.81E-01	9.84E-01	NO	
P07724	Alb	-0.0054	9.87E-01	9.87E-01	YES	secreted

Supplementary Table 2. Significantly altered liver amino acids and small metabolites in PiZ RΔhep compared to all other groups.

Name	Adjusted global p-value
Symmetric dimethylarginine	0.00026957790512395
Putrescine	0.00035331890913052
Total dimethylarginine	0.00053932069353682
Tyrosine	0.00135458132811021
Alanine	0.00259973386336734
Glutamic acid	0.00301501495606332
Total amino acids	0.00396839565856110
Non-essential amino acids	0.00396839565856110
Aromatic amino acids	0.00479321437980767
Histidine	0.00519432700353877
Glucogenic amino acids	0.00575602234100191
Spermidine./.putrescine	0.00745110037719596
Arginine	0.01530822617330570
Asymmetric dimethylarginine	0.02448719931702960
Methionine sulfoxide	0.03632963674798140
Spermidine	0.03724399979007750
Creatinine	0.04010984443034250

Supplementary Table 3. Genotyping and qRT-PCR oligonucleotides

Gene	Abbreviation	5'- Sequence - 3'
**Oligonucleotides for genotyping**		
AlbCre	Cre+	F: GCCAGCTAAACATGCTTCATC<br>R: ATTGCCCCTGTTTCACTATCC
CHOP	CHOP	F: ATGCCCTTACCTATCGTG<br>R1: GCAGGGTCAAGAGTAGTG<br>R2: AACGCCAGGGTTTTCCCAGTCA
Mtor	mTOR	F: TTATGTTTGATAATTGCAGTTTTGGCTAGCAGT<br>R: TTTAGGACTCCTTCTGTGACATACATTTCCT
Rictor	Rictor	F: CAAGCATCATGCAGCTCTTC<br>R: TCCCAGAATTTCCAGGCTTA
Rptor	Raptor	F: CTCAGTAGTGGTATGTGCTCAG<br>R: GGGTACAGTATGTCAGCACAG
SERPINA1	AAT	F: GCAGCCTGACTTCTTTGTGC<br>R: ATCCTAGGGGGCTTGGTGAT
**Primers for qRT-PCR**		
Ribosomal protein L7	L7	F: GAAAGGCAAGGAGGAAGCTCATCT<br>R: AATCTCAGTGCGGTACATCTGCCT
SERPINA1	AAT	F: TGCTTAAATACGGACGAGGA<br>R: ATTGTCGATTCACTGTCCCA
Ho1		F: GAATCGAGCAGAACCAGCCT<br>R:GCCTTCTCTGGACACCTGAC
Noq1		F: TGACATCACAGGTGAGCTG<br>R: ACCACTGCAATGGGAACTG
Cbr3		F: GGGACTTAACGTGCTGGTCA<br>R: GGGTGTTGGGTCATCCATTCT
CHOP		F: CCAGGAAACGAAGAGGAAGA<br>R: ATGTGCGTGTGACCTCTGTT

Supplementary Table 4. Antibodies used for immunoblotting, immunohistochemistry and immunofluorescence

Antibody	Company	Cat. #	Dilution
**Immunoblotting**			
β-Actin	Sigma	A1978	1:1000
AAT	Sigma	A0409	1:1000
BAX	Santa Cruz	sc-7480	1:1000
BIM	Cell Signalling	2933	1:1000
CHOP	Cell Signalling	5554	1:1000
EGFR	Cell Signalling	4267	1:1000
HGFR	Abcam	ab51067	1:1000
P62	Progen	GP62-C	1:1000
P-JNK	Cell Signaling	4668	1:1000
Grp78 (Bip)	Santa cruz	sc-756	1:500
OTC	Abcam	ab203859	1:1000
GAPDH	Novusbio	NB300-221	1:1000
Goat anti-rabbit IgG (H+L), (HRP conjugated)	Invitrogen	G21234	1:5000
Goat anti-mouse IgG (H+L), (HRP conjugated)	Invitrogen	G21040	1:5000
**Immunohistochemistry and immunofluorescence**			
ARG1	Abcam	ab91279	1:200
Caspase-3	Cell Signalling	9661	1:200
CHOP	Cell Signalling	5554	1:200
GS	Abcam	ab228590	1:100
CYP2E1	Abcam	ab29146	1:1000
Ki67	DAKO	GA626	1:200
CD45	Cell Signaling	70257	1:400
F4/80	Bio-Rad	MCA497	1:200
Clec4f	R&D Systems	AF2784	1:1000
Biotinylated goat anti-rabbit IgG antibody	Vector	BA-1000	1:200
Alexa Fluor 488 Goat Anti-rabbit IgG (H+L)	Invitrogen	A11034	1:200
Alexa Fluor 568 Goat Anti-rabbit IgG (H+L)	Invitrogen	A11036	1:200
RAPTOR	Cell Signalling	2280	1:1000
RICTOR	Cell Signalling	2114	1:1000
**Spatial Proteomics**			
Anti-glutamine synthase	Abcam	ab176562	1:100
Anti-rabbit nanobody coupled to Alexa Fluor 647	Chromotek	srbAF647-1-100	1:500

Antibody	Company	Cat. #	Dilution
Anti-E-cadherin coupled to Alexa Fluor 555	BD Biosciences	560064	1:100
Phalloidin coupled to AlexaFluor 488	Life technologies	A12379	1:300
